# Hard ticks (Acari, Ixodidae) of Croatia

**DOI:** 10.3897/zookeys.234.3658

**Published:** 2012-10-30

**Authors:** Stjepan Krčmar

**Affiliations:** 1 Department of Biology, J.J. Strossmayer University of Osijek, Cara Hadrijana 8/A, HR-31000 Osijek, Croatia

**Keywords:** Ixodidae, hard ticks, Croatia, Europe

## Abstract

The present paper is based on original and literature data. In Croatia the first studies on the occurrence of ixodid species were made about 80 years ago. The number of tick species recorded in Croatia considerably increased during the 1950s, 60s, 70s and 80s of the past century. A total of 21 species of hard tick belonging to 5 genera have been recorded in Croatia. *Ixodes* is the best represented genus, with seven species recorded. *Haemaphysalis* is represented by six species, followed by *Rhipicephalus* with four species. *Dermacentor* and *Hyalomma* are represented by two species each. The ticks were collected on 47 different host species. Eleven tick species were collected on *Bos taurus* and *Ovis aries*, followed by *Capra hircus* and *Equus caballus* with 8 species and *Canis lupus familiaris* with 6 species. On the remaining 42 host species one, two or three tick species were collected. The most widespread tick is *Ixodes ricinus* which was found on 25 different host species.

## Introduction

Hard ticks (Acari: Ixodidae) are obligate hematophaguos ectoparasites and important vectors of viruses, bacteria and protozoa (Rijpkema et al. 1996, Polić et al. 2002, Duh et al. 2006, 2010, Hornok and Farkas 2009, Hubálek 2010). They are considered second only to mosquitoes as the most medically important group of arthropods (Jaenson and Jensen 2007). Approximately 10% of the currently known 896 tick species are vectors of pathogens from wild animals to humans (Milutinović and Radulović 2002). In Croatia, the first comprehensive studies on the occurrence of ixodid species were made about 80 years ago (Oswald 1940, 1941a, 1941b). During the 1950s and 1960s, ticks in Croatia were studied from the ecological point of view (Mikačić 1949, 1961, 1963, 1965, 1968, 1969). For that period, the data on tick fauna along the Adriatic Coast are numerous because Prof. Dr. Davor Mikačić, in several of his articles, presented the distribution and dynamics of hard ticks along the Adriatic coast from Pula to Dubrovnik. Furthermore, ecological studies of ticks were carried out on many islands in the Adriatic Sea during the 1970s, and 1980s, with special emphasis on the medical importance of ticks (Tovornik 1976a, 1976b, 1980, 1984, 1987a, 1988b, 1990a, 1991a, 1991b, Tovornik and Černy 1972, Tovornik and Šooš 1976, Tovornik and Brelih 1980, Tovornik and Vesenjak-Hirjan 1988, 1989, Vesenjak-Hirjan 1976, Vesenjak-Hirjan et al. 1977). In Europe, the number of papers on canine tick-borne diseases has progressively increased in the past few years (Földvári 2005). Climate change contributes significantly to the change of habitat conditions which may affect the geographic range expansion of some species of ticks and thus the spread of various pathogens (Lindgren et al. 2000). This indicates the importance of creating a list of the tick fauna in European countries, due to possible changes in the fauna of ticks and tick-borne pathogens. Another reason for creating a list of the Croatian tick fauna is that some tick species have been mistakenly referred to as new for the fauna or were omitted from the list of the Croatian tick fauna on the website of Fauna Europaea, although they had already been identified many years ago. Thus, the principal aim of this study is to summarize all available data on the tick fauna of Croatia. This work is based on literature findings and on the data obtained from faunistic research carried out during 2011 in the continental part of eastern Croatia (Slavonia and Baranja).

## Material and methods

The following lists of species include all available literary records and new, still unpublished data. The samplings of ticks in Slavonia and Baranja were carried out during 2011 from the beginning of March to mid-September. Ticks were sampled by the flagging method, and were picked by hand from domestic animals and humans. The flagging method was used for tick sampling in two different vegetation types around the village of Mikleuš (YL 15). A 1 m^2^ white flag was dragged over the vegetation for about 50 m at each locality. The first locality was in the mixed forests of durmast oak and hornbeam (as. *Epimedio-Carpinetum betuli* /Horvat 1938/ Borhidi 1963), whereas the second locality was in open biotopes on grassland. In the veterinary clinic in Đakovo (BR 92), ticks were sampled from dogs during vaccination or during normal veterinary examinations, while sampling from cats was performed in the village of Zmajevac (CR 37). Also, some ticks were sampled from humans in the village of Zmajevac. Identification was carried out using standard keys for European ticks (Hillyard 1996, Estrada-Peña et al. 2004). The names of the species were written according to Guglielmone et al. (2010). The nomenclature of hosts follows Fauna Europaea (http://www.faunaeur.org ) and ITIS (Integrated Taxonomic Information System), (http://www.itis.usda.gov/index.html ) for livestock.

## A list of the host species and ticks collected on them

Phylum: Chordata

Subphylum: Vertebrata

Class: Mammalia

Order: Artiodactyla

Family: Bovidae

Genus: *Bos* Linnaeus, 1758

***Bos taurus*** Linnaeus, 1758

Tick species: *Dermacentor marginatus* (Sulzer, 1776), *Dermacentor reticulatus* (Fabricius, 1794), *Haemaphysalis punctata* Canestrini and Fanzago, 1878, *Haemaphysalis inermis* Birula, 1895, *Hyalomma scupense* Schulze, 1919, *Hyalomma marginatum* Koch, 1844, *Rhipicephalus annulatus* (Say, 1821), *Rhipicephalus bursa* Canestrini and Fanzago, 1878, *Rhipicephalus sanguineus* (Latreille, 1806), *Ixodes hexagonus* Leach, 1815, *Ixodes ricinus* (Linnaeus, 1758)

Genus: *Capra* Linnaeus, 1758

***Capra hircus*** (Linnaeus, 1758)

Tick species: *Dermacentor marginatus*, *Haemaphysalis punctata*, *Haemaphysalis sulcata* Canestrini and Fanzago, 1878, *Hyalomma marginatum*, *Rhipicephalus bursa*, *Rhipicephalus sanguineus*, *Rhipicephalus turanicus* Pomerantsev, 1940, *Ixodes ricinus*

Genus: *Ovis* Linnaeus, 1758

***Ovis aries*** Linnaeus, 1758

Tick species: *Dermacentor marginatus*, *Haemaphysalis punctata*, *Haemaphysalis inermis*, *Haemaphysalis sulcata*, *Hyalomma scupense*, *Hyalomma marginatum*, *Rhipicephalus bursa*, *Rhipicephalus sanguineus*, *Rhipicephalus turanicus*, *Ixodes gibbosus* Nuttal, 1916, *Ixodes ricinus*

Family: Cervidae

Genus: *Capreolus* Gray, 1821

***Capreolus capreolus*** (Linnaeus, 1758)

Tick species: *Haemaphysalis concinna* Koch, 1844, *Rhipicephalus turanicus*, *Ixodes ricinus*

Order: Carnivora

Family: Canidae

Genus: *Canis* Linnaeus, 1758

***Canis aureus*** (Linnaeus, 1758)

Tick species: *Rhipicephalus sanguineus*

***Canis lupus familiaris*** Linnaeus, 1758

Tick species: *Dermacentor marginatus*, *Haemaphysalis inermis*, *Rhipicephalus sanguineus*, *Rhipicephalus turanicus*, *Ixodes hexagonus*, *Ixodes ricinus*

Family: Mustelidae

Genus: *Martes* Pinel, 1792

***Martes foina*** (Erxleben, 1777)

Tick species: *Ixodes frontalis* (Panzer, 1798)

***Martes martes*** (Linnaeus, 1758)

Tick species: *Ixodes hexagonus*, *Ixodes ricinus*

Family: Felidae

Genus: *Felis* Linnaeus, 1758

***Felis catus*** Linnaeus, 1758

Tick species: *Dermacentor marginatus*, *Rhipicephalus turanicus*, *Ixodes ricinus*

Order: Chiroptera

Family: Rhinolophidae

Genus: *Rhinolophus* Lacepede, 1799

***Rhinolophus ferrumequinum*** (Schreiber, 1774)

Tick species: *Ixodes vespertilionis* Koch, 1844

***Rhinolophus hipposideros*** (Bechstein, 1800)

Tick species: *Ixodes vespertilionis*

Family: Vespertilionidae

Genus: *Miniopterus* Bonaparte, 1837

***Miniopterus schreibersii*** (Kuhl, 1817)

Tick species: *Ixodes vespertilionis*

Genus: *Myotis* Kaup, 1829

***Myotis myotis*** (Borkhausen, 1797)

Tick species: *Ixodes vespertilionis*

Order: Insectivora

Family: Erinaceidae

Genus: *Erinaceus* Linnaeus, 1758

***Erinaceus concolor*** Martin, 1838

Tick species: *Ixodes hexagonus*

***Erinaceus europaeus*** Linnaeus, 1758

Tick species: *Hyalomma marginatum*

Family: Soricidae

Genus: *Neomys* Kaup, 1829

***Neomys*** sp.

Tick species: *Ixodes trianguliceps* Birula, 1895

Genus: *Sorex* Linnaeus, 1758

***Sorex alpinus*** Schinz, 1837

Tick species: *Ixodes trianguliceps*

***Sorex araneus*** Linnaeus, 1758

Tick species: *Ixodes trianguliceps*

Order: Perissodactyla

Family: Equidae

Genus: *Equus* Linnaeus, 1758

***Equus asinus*** Linnaeus, 1758

Tick species: *Rhipicephalus sanguineus*, *Ixodes ricinus*

***Equus caballus*** Linnaeus, 1758

Tick species: *Dermacentor marginatus*, *Dermacentor reticulatus*, *Haemaphysalis punctata*, *Hyalomma marginatum*, *Rhipicephalus annulatus*, *Rhipicephalus bursa*, *Ixodes hexagonus*, *Ixodes ricinus*

Order: Rodentia

Family: Muridae

Genus: *Apodemus* Kaup, 1829

***Apodemus agrarius*** (Pallas, 1771)

Tick species: *Dermacentor marginatus*, *Ixodes ricinus*, *Ixodes trianguliceps*

***Apodemus flavicollis*** (Melchior, 1834)

Tick species: *Ixodes ricinus*, *Ixodes trianguliceps*

***Apodemus mystacinus*** (Danford et Alston, 1877)

Tick species: *Ixodes ricinus*, *Ixodes trianguliceps*

***Apodemus sylvaticus*** (Linnaeus, 1758)

Tick species: *Dermacentor marginatus*, *Ixodes ricinus*, *Ixodes trianguliceps*

Genus: *Clethrionomys* Tilesius, 1850

***Clethrionomys glareolus*** (Schreber, 1780)

Tick species: *Dermacentor marginatus*, *Ixodes ricinus*, *Ixodes trianguliceps*

Genus: *Microtus*, Schrank, 1798

***Microtus arvalis*** (Pallas, 1779)

Tick species: *Dermacentor marginatus*

Order: Primates

Family: Hominidae

Genus: *Homo* Linnaeus, 1758

***Homo sapiens*** Linnaeus, 1758

Tick species: *Ixodes ricinus*

Class: Aves

Order: Apodiformes

Family: Apodidae

Genus: *Tachymarptis* (Linnaeus, 1758)

***Tachymarptis melba*** (Linnaeus, 1758)

Tick species: *Haemaphysalis erinacei* Pavesi, 1884

Order: Falconiformes

Family: Accipitridae

Genus: *Buteo* Lacepede, 1799

***Buteo buteo*** (Linnaeus, 1758)

Tick species: *Ixodes ricinus*

Order: Passeriformes

Family: Corvidae

Genus: *Pica* Brisson, 1760

***Pica pica*** (Linnaeus, 1758)

Tick species: *Ixodes ricinus*

Family: Bombycillidae

Genus: *Bombycilla* Vieillot, 1808

***Bombycilla garrulus*** (Linnaeus, 1758)

Tick species: *Ixodes frontalis*

Family: Saxicolidae

Genus: *Luscinia* Forster, 1817

***Luscinia megarhynchos*** C.L. Brehm 1831

Tick species: *Ixodes ricinus*

Genus: *Phoenicurus* Forster, 1817

***Phoenicurus ochruros*** (S. G. Gmelin, 1774)

Tick species: *Ixodes frontalis*

Family: Turdidae

Genus: *Turdus* Linnaeus, 1758

***Turdus merula*** Linnaeus, 1758

Tick species: *Ixodes arboricola* Schulze and Schlottke, 1930, *Ixodes ricinus*

***Turdus philomelos*** C. L. Brehm, 1831

Tick species: *Ixodes ricinus*

Family: Motacillidae

Genus: *Anthus* Bechstein, 1805

***Anthus trivialis*** (Linnaeus, 1758)

Tick species: *Ixodes ricinus*

Genus: *Motacilla* Linnaeus, 1758

***Motacilla flava*** Linnaeus, 1758

Tick species: *Haemaphysalis punctata*

Family: Sylviidae

Genus: *Cettia* Bonaparte, 1834

***Cettia cetti*** (Temminck, 1820)

Tick species: *Ixodes ricinus*

Class: Reptilia

Order: Squamata

Family: Lacertidae

Genus: *Lacerta* Linnaeus, 1758

***Lacerta bilineata*** Daudin, 1802

Tick species: *Haemaphysalis concinna*

***Lacerta trilineata*** Bedriaga, 1886

Tick species: *Haemaphysalis sulcata*

***Lacerta viridis*** (Laurenti, 1768)

Tick species: *Haemaphysalis sulcata*

Genus: *Dalmatolacerta* Arnold, Arribas & Carranza, 2007

***Dalmatolacerta oxycephala*** (Duméril & Bibron, 1839)

Tick species: *Haemaphysalis sulcata*

Genus: *Podarcis* Wagler, 1830

***Podarcis melisellensis*** (Braun, 1877)

Tick species: *Haemaphysalis concinna*, *Haemaphysalis sulcata*, *Ixodes ricinus*

***Podarcis muralis*** (Laurenti, 1768)

Tick species: *Haemaphysalis concinna*

***Podarcis sicula*** (Rafinesque, 1810)

Tick species: *Haemaphysalis sulcata*, *Ixodes ricinus*

Genus: *Algyroides* (Bibron & Bory de Saint-Vincent, 1833)

***Algyroides nigropunctatus*** (Duméril & Bibron, 1839)

Tick species: *Haemaphysalis concinna*, *Haemaphysalis sulcata*, *Ixodes ricinus*

Family: Colubridae

Genus: *Elaphe* Fitzinger, 1833

***Elaphe longissima*** (Laurenti, 1768)

Tick species: *Haemaphysalis concinna*

## Sampling sites (localities)

**Figure 1.**
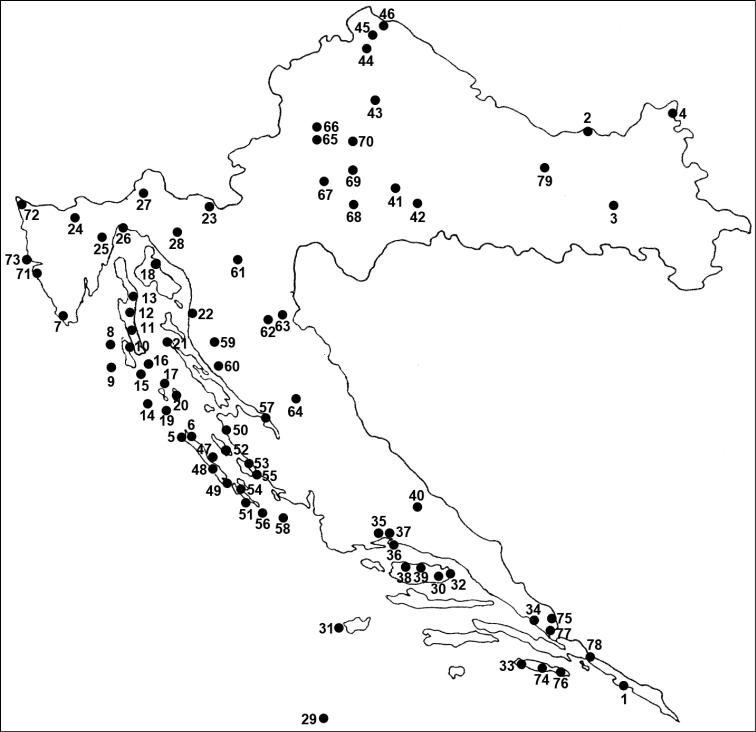
Sampling sites of hard ticks (Ixodidae) in Croatia.

Sampling sites are identified by a running number referring to the map in Figure 1 and UTM grid 10×10 km given in the third column of Table 1. The exact geographical coordinates of the sampling sites are given in the second column of Table 1 and were determined by (http://www.google.com/earth/index.html , http://www.worldplaces.net , http://www.plsavez.hr ).

**Table 1.** List of sampling sites

	**Locality**	**Altitude-latitude<br/> (degree/min/sec)**	**UTM**
1.	Dubrovnik<br/> Močiljska špilja<br/> Lokrum Island	42°39'02"N, 18°05'40"E<br/> 42°41'17"N, 18°04'24"E<br/> 42°37'52"N, 18°07'03"E	BN 62
2.	Kapelački Lug	45°45'37"N, 18°09'53"E	BR 87
3.	Đakovo	45°18'25"N, 18°25'01"E	BR 92
4.	Zmajevac	45°47'59"N, 18°48'23"E	CR 37
5.	Vrtlac reef	43°37'26"N, 15°36'31"E	VJ 88
6.	Baričevac Island<br/> Golac Island<br/> Mežanj Island<br/> Sparušnjak Island	44°09'12"N, 14°51'02"E<br/> 44°11'16"N, 14°50'44"E<br/> 44°05'39"N, 14°55'07"E<br/> 44°08'12"N, 14°57'06"E	VJ 98
7.	Pula	44°51'59"N, 13°50'58"E	VK 16
8.	Unije Island	44°37'29"N, 14°15'34"E	VK 34
9.	Susak Island<br/> Vele Srakane Island	44°30'28"N, 14°18'14"E<br/> 44°34'51"N, 14°18'41"E	VK 42
10.	Trstenik Island<br/> Školjić reef	44°40'06"N, 14°34'46"E<br/> 44°38'09"N, 14°13'54"E	VK 54
11.	Cres Island: Belej<br/> Hrasta<br/> Zeča Island	44°46'41"N, 14°25'46"E<br/> 44°48'50"N, 14°25'14"E<br/> 44°46'20"N, 14°18'41"E	VK 56
12.	Cres Island: Cres<br/> Orlec<br/> Vrana	44°57'38"N, 14°24'33"E<br/> 44°51'57"N, 14°27'06"E<br/> 44°50'40"N, 14°26'32"E	VK 57
13.	Cres Island: Petrićevi- Špilja Čampari<br/> Vodice	45°07'06"N, 14°19'51"E<br/> 45°00'31"N, 14°23'58"E	VK 58
14.	Kamenjak Island<br/> Lutrošnjak Island<br/> Premuda Island<br/> Južni Greben Island<br/> Zapadni Greben Island	44°21'24"N, 14°34'49"E<br/> 44°21'51"N, 14°34'26"E<br/> 44°20'39"N, 14°35'56"E<br/> 44°19'05"N, 14°42'41"E<br/> 44°19'46"N, 14°41'42"E	VK 61
15.	Grujica Island<br/> Ilovik Island<br/> Male Orjule Island<br/> Sveti Petar Island	44°24'37"N, 14°34'09"E<br/> 44°27'09"N, 14°33'05"E<br/> 44°29'20"N, 14°33'53"E<br/> 44°27'43"N, 14°33'29"E	VK 62
16.	Lošinj Island: Veli Lošinj,<br/> Oruda Island,<br/> Palacol Island<br/> Ćunski-Like	44°31'09"N, 14°30'09"E<br/> 44°33'07"N, 14°34'57"E<br/> 44°32'40"N, 14°35'39"E<br/> 44°35'21"N, 14°24'35"E	VK 63
17.	Morovnik Island<br/> Silba Island<br/> Kurjak reef	44°25'55"N, 14°44'03"E<br/> 44°24'02"N, 14°41'03"E<br/> 44°24'17"N, 14°45'06"E	VK 71
18.	Krk Island: Kozlja	45°00'38"N, 14°45'24"E	VK 79
19.	Dužac Island	44°15'09"N, 14°44'07"E	VK 80
20.	Olib Island<br/> Planičić reef<br/> Šip reef	44°22'38"N, 14°47'09"E<br/> 44°21'45"N, 14°52'31"E<br/> 44°25'06"N, 14°45'16"E	VK 81
21.	Mali Dolfin reef<br/> Mali Laganj Island	44°41'22"N, 14°41'31"E<br/> 44°42'49"N, 14°39'59"E	VK 93
22.	Velebit mountain:<br/> Donja Klada<br/> Veliki Zavižan	44°48'52"N, 14°53'05"E<br/> 44°48'09"N, 14°58'25"E	VK 96
23.	Lukovdol- Lipov Vrh	45°25'33"N, 15°07'37"E	VL 03
24.	Buzet-Rabakova Peć<br/> Ročko Polje	45°24'27"N, 13°57'54"E<br/> 45°22'11"N, 14°04'44"E	VL 22
25.	Istra: Učka mountain	45°17'01"N, 14°14'58"E	VL 31
26.	Rijeka	45°19'40"N, 14°26'32"E	VL 51
27.	Gorski Kotar: Risnjak mountain	45°25'54"N, 14°37'17"E	VL 63
28.	Sungerski Lug	45°19'22"N, 14°49'12"E	VL 81
29.	Kamik od Tramuntane<br/> Palagruža Island	42°23'20"N, 16°16'30"E<br/> 42°23'33"N, 16°15'32"E	XG 09
30.	Brač Island: Blato<br/> Nadsela<br/> Osridke<br/> Sveti Toma	43°17'02"N, 16°40'41"E<br/> 43°17'16"N, 16°48'49"E<br/> 43°16'55"N, 16°49'24"E<br/> 43°17'34"N, 16°47'00"E	XH 49
31.	Brusnik Island<br/> Pod Mrčaru reef	43°00'29"N, 15°47'55"E<br/> 42°46'47"N, 16°46'30"E	XH 43
32.	Brač Island:<br/> Zapadna Laščatna<br/> Sjeverna Laščatna	<br/> 43°19'02"N, 16°53'14"E<br/> 43°19'01"N, 16°53'25"E	XH 59
33.	Mljet Island: Soline<br/> Veliko Jezero	42°46'04"N, 17°22'55"E<br/> 42°46'30"N, 17°21'40"E	XH 93
34.	Baćinska jezera	43°04'39"N, 17°24'54"E	XH 97
35.	Kaštela, Kozjak mountain:<br/> Malačka	43°33'51"N, 16°21'29"E<br/> 43°34'44"N, 16°21'04"E	XJ 02
36.	Split	43°30'29"N, 16°26'25"E	XJ 11
37.	Kaštela Bay	43°33'09"N, 16°20'36"E	XJ 12
38.	Brač Island: Supetar	43°23'00"N, 16°33'23"E	XJ 20
39.	Brač Island	43°18'17"N, 16°39'09"E	XJ 30
40.	Sinj	43°42'10"N, 16°38'15"E	XJ 34
41.	Srednja Posavina: Stružec	45°31'37"N, 16°32'59"E	XL 24
42.	Repušnica	45°29'44"N, 16°43'50"E	XL 33
43.	Križevci<br/> Stara Ves	45°59'04"N, 16°34'29"E<br/> 45°58'35"N, 16°29'56"E	XL 39
44.	Nedelišće	46°22'34"N, 16°23'08"E	XM 12
45.	Čakovec	46°23'24"N, 16°26'17"E	XM 13
46.	Dekanovec	46°26'48"N, 16°34'49"E	XM 24
47.	Maslinovac Island	42°55'11"N, 17°29'30"E	WJ 07
48.	Mali Prišnjak<br/> Malo Šilo Island<br/> Mrtovac Island	43°50'49"N, 15°33'34"E<br/> 43°50'44"N, 15°14'10"E<br/> 43°49'39"N, 15°13'56"E	WJ 16
49.	Božikovac Island<br/> Mala Lavdara Island<br/> Mala Skala Island<br/> Rončić Island<br/> Trimulić Island<br/> Trstikovac Island<br/> Vela Skala Island	43°55'57"N, 15°15'09"E<br/> 43°55'00"N, 15°13'48"E<br/> 43°54'33"N, 15°15'54"E<br/> 43°54'20"N, 15°16'26"E<br/> 43°54'29"N, 15°14'40"E<br/> 43°54'31"N, 15°14'56"E<br/> 43°54'56"N, 15°15'38"E	WJ 17
50.	Kali<br/> Vela Sestrica<br/> Zadar	44°03'49"N, 15°12'23"E<br/> 44°11'15"N, 14°59'21"E<br/> 44°07'11"N, 15°13'59"E	WJ 18
51.	Veseljuh Island	43°45'52"N, 15°21'55"E	WJ 25
52.	Gušteranski Island<br/> Koromašna Island<br/> Kosmerka Island<br/> Škrovada reef	43°38'43"N, 15°42'48"E<br/> 43°39'24"N, 15°41'06"E<br/> 43°38'00"N, 15°36'12"E<br/> 43°37'36"N, 15°40'47"E	WJ 26
53.	Dugi otok Island<br/> Pašman Island	43°57'18"N, 15°05'47"E<br/> 43°58'18"N, 15°19'46"E	WJ 27
54.	Bisaga Island<br/> Mali Babuljaš Island<br/> Veli Babuljaš Island	43°48'29"N, 15°17'00"E<br/> 43°48'02"N, 15°17'16"E<br/> 43°47'57"N, 15°17'02"E	WJ 35
55.	Biograd-Vrana	43°56'09"N, 15°26'33"E	WJ 36
56.	Babina Guzica Island<br/> Jančar Island<br/> Kameni Puh Island<br/> Mala Prduša Island<br/> Purara Island<br/> Veli Puh Island<br/> Vodeni Puh Island<br/> Vodenjak Island	43°42'36"N, 15°29'52"E<br/> 43°43'09"N, 15°25'52"E<br/> 43°40'38"N, 15°30'42"E<br/> 43°42'33"N, 15°27'11"E<br/> 43°41'47"N, 15°26'14"E<br/> 43°40'31"N, 15°29'45"E<br/> 43°40'36"N, 15°31'05"E<br/> 43°43'42"N, 15°23'50"E	WJ 43
57.	Rovanjska	44°14'52"N, 15°32'31"E	WJ 49
58.	Čavlin Island<br/> Čerigul Island<br/> Mali Kamešnjak Island<br/> Mali Tetovišnjak Island<br/> Mikavica Island<br/> Samograd Island<br/> Veli Kamešnjak Island<br/> Veli Tetovišnjak Island<br/> Žirje Island	43°44'03"N, 15°34'54"E<br/> 43°43'39"N, 15°36'39"E<br/> 43°40'19"N, 15°42'02"E<br/> 43°43'07"N, 15°36'08"E<br/> 43°40'44"N, 15°36'53"E<br/> 43°41'16"N, 15°33'26"E<br/> 43°40'21"N, 15°41'29"E<br/> 43°43'24"N, 15°35'37"E<br/> 43°39'02"N, 15°39'32"E	WJ 53
59.	Velebit mountain: Crni Padež	44°40'18"N, 15°03'06"E	WK 04
60.	Velebit mountain: Baške Oštarije	44°31'51"N, 15°10'51"E	WK 12
61.	Velika Kapela mountain: Razvala	45°04'35"N, 15°12'41"E	WK 29
62.	Lika: Plitvice-Crna rijeka	44°50'41"N, 15°37'45"E	WK 46
63.	Lika: Plitvice-Mukinje	44°52'27"N, 15°37'45"E	WK 56
64.	Velebit mountain: Prezid, Pod ćelavcem	44°15'07"N, 15°50'12"E	WK 60
65.	Zagreb	45°48'47"N, 15°58'40"E	WL 77
66.	Zagreb- vicinity, Medvednica mountain: Sljeme	45°53'58"N, 15°57'09"E	WL 78
67.	Vukomeričke Gorice: Prkovec	45°37'00"N, 15°56'59"E	WL 84
68.	Petrinja	45°25'31"N, 16°14'48"E	WL 93
69.	Peščenica-Vratovo	45°36'08"N, 16°10'05"E	WL 95
70.	Dugo Selo	45°48'21"N, 16°14'16"E	WL 97
71.	Banjol Island<br/> Figarola Island<br/> Gustinja Island<br/> Pisulj Island<br/> Pulari Island<br/> Revera Island<br/> Rovinj<br/> Sturag Island<br/> Sveti Ivan Island<br/> Sveti Ivan np reef<br/> Velika Sestrica Island<br/> Veliki Piruzi reef<br/> Veštar Island	45°04'21"N, 13°36'35"E<br/> 45°05'41"N, 13°37'17"E<br/> 45°00'59"N, 13°40'53"E<br/> 45°01'12"N, 13°41'51"E<br/> 45°03'25"N, 13°39'51"E<br/> 45°03'02"N, 13°39'34"E<br/> 45°04'52"N, 13°38'19"E<br/> 45°03'05"N, 13°37'34"E<br/> 45°02'49"N, 13°37'17"E<br/> 45°02'33"N, 13°36'50"E<br/> 45°02'00"N, 13°40'28"E<br/> 45°03'32"N, 13°38'22"E<br/> 45°02'51"N, 13°40'42"E	UK 99
72.	Umag	45°26'02"N, 13°38'22"E	UL 83
73.	Koversada<br/> Lakal Island<br/> Lunga Island<br/> Tovarjež Island	45°08'03"N, 13°35'51"E<br/> 45°09'41"N, 13°35'33"E<br/> 45°08'32"N, 13°34'58"E<br/> 45°10'07"N, 13°34'55"E	UL 90
74.	Mljet Island:<br/> Babino Polje<br/> Blato<br/> Zadublje	<br/> 42°44'08"N, 17°33'03"E<br/> 42°45'45"N, 17°28'52"E<br/> 42°44'50"N, 17°33'25"E	YH 03
75.	Metković	43°03'12"N, 17°38'57"E	YH 06
76.	Mljet Island	42°44'38"N, 17°32'19"E	YH 13
77.	Metković-Gabela	43°04'01"N, 17°39'08"E	YH 16
78.	Slano	42°47'13"N, 17°53'33"E	YH 33
79.	Mikleuš	45°36'46"N, 17°48'33"E	YL 15

## Results

The tick fauna of Croatia is now known to include 21 species. These species are classified into five genera: *Dermacentor* Koch, 1844, *Haemaphysalis* Koch, 1844, *Hyalomma* Koch, 1844, *Rhipicephalus* Koch, 1844, and *Ixodes* Latreille, 1795. The genera *Dermacentor* and *Hyalomma* are represented by a two species, followed by *Rhipicephalus* with four species, *Haemaphysalis* with six species,and *Ixodes* with seven species. While studying the territory of Slavonia and Baranja during 2011, a total of 1425 ticks were collected. All the collected ticks were classified into 5 species. The identified species were classified into the following genera: *Dermacentor*, *Haemaphysalis* and *Ixodes*. *Ixodes ricinus* made up 83.44% of the tick fauna in the researched area, while 16.56% were representatives of other species.

## Survey of hard ticks recorded in Croatia

For every species are mentioned, sex, developmental stage ( ♂ male, ♀ female N = nymph, L = larva), sampling place, month or date, host and data source.

**Family IXODIDAE Murray, 1877**

**Genus** *Dermacentor* Koch 1844

**1. *Dermacentor marginatus* (Sulzer, 1776)**

♂♂,♀ ♀ Cres Island: Hrasta 10.XI.1948, from *Ovis aries* (Mikačić 1949)

♂♂,♀ ♀ ibidem: Vodice 6.–11.IV. 1948, from *Ovis aries* (Mikačić 1965)

♂♂,♀♀ ibidem: Hrasta 29.IX.–1.X.1948, from *Ovis aries* (Mikačić 1965)

♂♂,♀♀ ibidem: Belej 29.IX.–1.X.1948, 4.XI. 1948, from *Ovis aries* (Mikačić 1965)

♂♂, ♀♀ Pula, months: I. II. III. IV.V. IX. X. XI. XII. 1936–1963 from *Ovis aries*, *Equus caballus*, *Bos taurus* (Mikačić 1965)

♂♂, ♀♀ Krk Island, months: I. II. IX. X. XI. XII. 1936–1963 from *Ovis aries*, *Equus caballus*, *Bos taurus* (Mikačić 1965)

♂♂, ♀♀ Cres Island, months: I. II. III. IV. VIII. IX. X. XI. XII. 1936–1963 from *Ovis aries*, *Equus caballus*, *Bos taurus* (Mikačić 1965)

♂♂, ♀♀ Lošinj Island: Veli Lošinj, months: I. II. III. IV. VIII. IX. X. XII. 1936–1963 from *Ovis aries*, *Equus caballus*, *Bos taurus* (Mikačić 1965)

♂♂,♀♀ ibidem: Čunski-Like, month: VIII. 1948, from *Ovis aries* (Mikačić 1965)

♂♂,♀ ♀ Zadar, months: I. II. III. IV.V.VI. IX. X. XI. XII. 1936–1963 from *Ovis aries*, *Equus caballus*, *Bos taurus* (Mikačić 1965)

♂♂, ♀♀ Kaštela, month: VI. 1936–1963 from *Ovis aries*, *Equus caballus*, *Bos taurus* (Mikačić, 1965)

♂♂, ♀♀ Split, months: I. II. III. IV.V.VI. XI. XII. 1936–1963 from *Ovis aries*, *Equus caballus*, *Bos taurus* (Mikačić 1965)

♂♂, ♀♀ Brač Island, months: I. II. III. IX. X. XI. XII. 1936–1963 from *Ovis aries*, *Equus caballus*, *Bos taurus* (Mikačić 1965)

♂♂, ♀♀ Dubrovnik, months: II. III. IV. IX. X. 1936–1963 from *Ovis aries*, *Equus caballus*, *Bos taurus* (Mikačić 1965)

♂♂, ♀♀ Srednja Posavina, months: III. IV. V. VI. XI. XII. from *Equus caballus*, *Bos taurus* (Mikačić 1968)

♂♂,♀♀ Brač Island: Sveti Toma 4.–9.XI. 1965 from *Ovis aries* (Tovornik and Černy 1972)

♂♂,♀♀ ibidem: Osridke 4.–9.XI.1965 from *Ovis aries* (Tovornik and Černy 1972)

♂♂,♀♀ ibidem: Blato 4.–9.XI. 1965 from *Ovis aries* (Tovornik and Černy 1972)

♂♂,♀♀ ibidem: months: I. II. X. XI. XII. 1964/1965 collected on vegetation using the cloth dragging method (Tovornik 1976b)

♂♂,♀♀ ibidem: Sveti Toma, months: I. II. XI. XII. 1964/1965 collected on vegetation using the cloth dragging method (Tovornik 1976b)

♂♂, ♀♀ ibidem: Zapadna Laščatna, months: I. X. XI. XII.1964/1965 collected on vegetation and/or the ground by flagging/dragging method (Tovornik 1976b)

♂♂, ♀♀ ibidem: Sjeverna Laščatna, months: XI. XII. 1964/1965 collected on vegetation using the cloth dragging method (Tovornik 1976b)

♂♂, ♀♀ ibidem: months: I. II. III. IX. X. XI. XII. 1964/1965 from *Ovis aries* (Tovornik 1976b)

NN ibidem: month:VII. 1964/1965 from *Ovis aries* (Tovornik 1976b)

♂♂, ♀♀, NN, LL Srednja Posavina: Stružec from 1970 to 1978 collected on vegetation using the cloth dragging method (Borčić et al. 1978)

NN Peščenica-Vratovo 17.VII. 1974 from *Microtus arvalis* (Tovornik 1988b)

NN Velebit mountain: Predzid (pod Ćelavcem) 1.VIII. 1975 from *Apodemus sylvaticus* (Tovornik 1988b)

♂♂,♀♀ Brač Island: Supetar 22.II. 1974 from *Ovis aries* (Tovornik 1991a)

L, NN Repušnica 5.,6.VIII. 1982 from *Clethrionomys glareolus* (Tovornik and Matjašič 1991)

N ibidem: 6.,7.VIII. 1982 from *Apodemus agrarius* (Tovornik and Matjašič 1991)

♂♂,♀♀ Čakovec, months: III. IV. V. 2007 collected on vegetation using the cloth-dragging method (Dobec et al. 2009)

♂♂, ♀♀ Kaštela Bay, month: X.2000 from *Ovis aries*, *Capra hircus*, *Bos taurus* (Punda -Polić et al. 2002)

♂♂,♀♀ Sinj, month: X. 2000, from *Ovis aries*, *Capra hircus*, *Bos taurus* (Punda-Polić et al. 2002)

♂♂,♀♀ Brač Island, Hvar Island (Hubálek 2010)

♂♂, ♀♀ Đakovo 10.III.–30.III. 2011 from *Canis lupus familiaris*

♂♂, ♀♀ ibidem: 7.IV.–25.IV. 2011 from *Canis lupus familiaris*

♂♂, ♀♀ ibidem: 2.V.–25.V.2011 from *Canis lupus familiaris*

♂, ♀ Zmajevac 15.III.- 30.III. 2011 from *Felis catus*

♀♀ ibidem: 10.IV.–24.IV. 2011 from *Felis catus*

♀♀ ibidem: 25.IV.–30.IV. 2011 from *Felis catus*

♀ ibidem: 7.V.–25.V.2011 from *Felis catus*

**2. *Dermacentor reticulatus* (Fabricius, 1794)**

♂♂, ♀♀ Gornja Posavina, months: III. IV. V. from *Equus caballus*, *Bos taurus* (Mikačić 1968)

♀ Kapelački Lug, month: V. 1967 collected on vegetation using the cloth dragging method (Vesenjak-Hirjan and Šooš 1976)

♂♂, ♀♀, NN, LL Srednja Posavina: Stružec -, from 1970 to 1978 collected on vegetation using the cloth dragging method (Borčić et al. 1978)

♂♂, ♀♀ Čakovec, months: III. IV. V. 2007 collected on vegetation using the cloth dragging method (Dobec et al. 2009)

♀♀ Mikleuš 27.IV.2011 collected on vegetation using the cloth dragging method

♀♀ ibidem: 14.V.2011 collected on vegetation using the cloth dragging method

**Genus** *Haemaphysalis* Koch, 1844

**3. *Haemaphysalis punctata* Canestrini & Fanzago, 1878**

♂♂, ♀♀ Cres Island: Hrasta 6.–11.IV.1948 from *Ovis aries* (Mikačić 1949)

♂♂, ♀♀ ibidem: 29.IX.–1.X. 1948 from *Ovis aries* (Mikačić 1949)

♂♂, ♀♀ ibidem: 10.XI. 1948 from *Ovis aries* (Mikačić 1949)

♂♂, ♀♀ ibidem: Vrana 6.–11.IV.1948 from *Capra hircus* (Mikačić 1949)

♂♂, ♀♀, N ibidem: Vodice 6.–11.IV.1948 from *Ovis aries* (Mikačić 1949)

♂♂, ♀♀ ibidem: Belej 29.IX.–1.X.1948 from *Ovis aries* (Mikačić 1949)

♂♂,♀♀ ibidem: 4.XI. 1948 from *Ovis aries* (Mikačić 1949)

♂♂, ♀♀ Lošinj Island: Veli Lošinj 29.IX.–1.X. 1948 from *Bos taurus* (Mikačić 1949)

♂♂, ♀♀ ibidem: Čunski-Like 29.IX.–1.X. 1948 from *Ovis aries* (Mikačić 1949)

♂♂, ♀♀, NN, LL Pula,months: I. II. , III. , IV. V. VI. , VII. , VIII. IX. , X. XI. XII. 1936–1963 from *Ovis aries*, *Equus caballus*, *Bos taurus* (Mikačić 1965)

♂♂, ♀♀ Krk Island,months: I. II. IV. IX. X. XI. XII. 1936–1963 from *Ovis aries*, *Equus caballus*, *Bos taurus* (Mikačić 1965)

♂♂, ♀♀ Cres Island,months: I. II. III. IV.V. IX. X. XI. XII. 1936–1963 from *Ovis aries*, *Equus caballus*, *Bos taurus* (Mikačić 1965)

♂♂, ♀♀ Lošinj Island: Veli Lošinj, months: I. II. III. IV. IX. X. XI. XII. 1936–1963 from *Ovis aries*, *Equus caballus*, *Bos taurus* (Mikačić 1965)

♂♂, ♀♀ Zadar,months: I. II. III. IV. V. VI. IX. X. XI. XII. 1936–1963 from *Ovis aries*, *Equus caballus*, *Bos taurus* (Mikačić 1965)

♂♂,♀♀, NN, LL Split,months: I. II. III. IV. X. XI. XII. 1936–1963 from *Ovis aries*, *Equus caballus*, *Bos taurus* (Mikačić 1965)

♂♂, ♀♀ Brač Island, months: II. III. VII. X. XI. 1936–1963 from *Ovis aries*, *Equus caballus*, *Bos taurus* (Mikačić 1965)

♂♂,♀♀ ibidem: months: I. II.III. IV. V. IX. X. XI. XII 1964/1965 from *Ovis aries* (Tovornik 1976b)

♂♂, ♀♀, NN Dubrovnik,months: I. II. III. IV.V. IX. X. XI. XII.1936–1963 from *Ovis aries*, *Equus caballus*, *Bos taurus* (Mikačić 1965)

♂♂, ♀♀ Gornja Posavina,month: V. from *Equus caballus*, *Bos taurus* (Mikačić 1968)

♂♂, ♀♀ Brač Island: Sveti Toma 4–9.XI.1965 from *Ovis aries* (Tovornik and Černy 1972)

♂♂, ♀♀ ibidem: Osridke 4.–9.XI. 1965 from *Ovis aries* (Tovornik and Černy 1972)

♂♂, ♀♀ ibidem: Blato 4.–9.XI. 1965 from *Ovis aries* (Tovornik and Černy 1972)

♂♂, ♀♀ ibidem: months: I. II. IV.V. XII. 1964/1965 collected on vegetation and/or the ground by flagging/dragging method (Tovornik 1976b)

♂♂, ♀♀ ibidem: Sveti Toma, months: I. II. IV.V. IX. X. XI. XII. 1964/1965 from *Ovis aries* (Tovornik 1976b)

♂♂, ♀♀ ibidem: Blato 5.XI.1965 from *Ovis aries* (Tovornik 1987a)

♂♂, ♀♀ ibidem: Zapadna Laščatna, months: I. IV. IX. X. XI. XII. 1964/1965 collected on vegetation and/or the ground by flagging/dragging method (Tovornik 1976b)

♂♂, ♀♀ ibidem: Sjeverna Laščatna, months: I. IV. XI. XII. 1964/1965 collected on vegetation and/or the ground by flagging/dragging method (Tovornik 1976b)

N Metković 23.IV. 1964 from *Motacilla flava* (Tovornik 1990a)

♂♂, ♀♀ Brač Island: Supetar 22.II. 1974 from *Ovis aries* (Tovornik 1991a)

♂♂, ♀♀ Kaštela Bay, month: X.2000 from *Ovis aries*, *Capra hircus*, *Bos taurus* (Punda -Polić et al. 2002)

♂♂, ♀♀ Sinj, month: X. 2000 from *Ovis aries*, *Capra hircus*, *Bos taurus* (Punda-Polić et al. 2002)

♂♂, ♀♀ Brač Island, Hvar Island (Hubálek 2010)

**4. *Haemaphysalis inermis* Birula, 1895**

♂♂, ♀♀ Cres Island, months: I. II.1936–1963 from *Ovis aries*, *Bos taurus*, *Canis lupus familiaris* (Mikačić 1965)

♂♂, ♀♀ Lošinj Island: Veli Lošinj, month: IX. 1936–1963 from *Ovis aries*, *Bos taurus*, *Canis lupus familiaris* (Mikačić 1965)

♂♂, ♀♀ Zadar, month: V. 1936–1963 from *Ovis aries*, *Bos taurus*, *Canis lupus familiaris* (Mikačić 1965)

♂♂, ♀♀ Dubrovnik, month: V. 1936–1963 from *Ovis aries*, *Bos taurus*, *Canis lupus familiaris* (Mikačić 1965)

**5. *Haemaphysalis concinna* Koch, 1844**

♂ Dekanovec 10.VI. 1977 from *Capreolus capreolus* (Tovornik 1988a)

NN Krk Island: 15.V.2002 from *Podarcis muralis*, *Podarcis melisellensis*,*Lacerta bilineata*, *Algyroides nigropunctatus*, *Elaphe longissima* (Hassl 2003)

♂♂ Mikleuš 14.V.2011 collected on vegetation using the cloth-dragging method

NN ibidem: 8.VI.2011 collected on vegetation using the cloth-dragging method

♀, NN ibidem: 9.VI.2011 collected on vegetation using the cloth-dragging method

♂ ibidem: 15.VI.2011 collected on vegetation using the cloth-dragging method

NN, LL ibidem: 3.VII.2011 collected on vegetation using the cloth-dragging method

♂, NN ibidem: 4.VII.2011 collected on vegetation using the cloth-dragging method

NN, LL ibidem: 2.VIII.2011 collected on vegetation using the cloth-dragging method

NN, LL ibidem: 3.VIII.2011 collected on vegetation using the cloth-dragging method

N ibidem: 13.IX.2011 collected on vegetation using the cloth-dragging method

**6. *Haemaphysalis sulcata* Canestrini & Fanzago, 1878**

♂♂, ♀♀ Pula, months: X. XI. 1936–1963 from *Ovis aries* (Mikačić 1965)

♂♂, ♀♀ Krk Island, months: IV. XI. XII. 1936–1963 from *Ovis aries* (Mikačić 1965)

♂♂, ♀♀ Cres Island, months: I. II. III. IV. V. X. XI. XII. 1936–1963 from *Ovis aries* (Mikačić 1965)

♂♂, ♀♀ Lošinj Island: Veli Lošinj, months: I. IV. X. 1936–1963 from *Ovis aries* (Mikačić 1965)

♂♂, ♀♀ Zadar, months: I. II. III. IV. XI. XII. 1936–1963 from *Ovis aries* (Mikačić 1965)

♂♂, ♀♀ Split, months: I. II. III. IV. 1936–1963 from *Ovis aries* (Mikačić 1965)

♂♂, ♀♀ Brač Island, months: I. II. III. IV. V. X. XI. XII. 1964/1965 from *Ovis aries* (Tovornik 1976b)

NN ibidem: months: VII. VIII. 1964/1965 from *Ovis aries* (Tovornik 1976b)

♀ ibidem: Nadsela 1.X.1965 from *Ovis aries* (Tovornik 1987a)

L Krk Island: 23.VII.1956 from *Algyroides nigropunctatus* (Tovornik and Brelih 1980)

L ibidem: 23.VII.1956 from *Podarcis melisellensis* (Tovornik and Brelih 1980)

N Cres Island: Vrana, month: VII.1949 from *Lacerta trilineata* (Tovornik and Brelih 1980)

NN, LL Trstenik Island: 20.VII.1965 from *Lacerta viridis* (Tovornik and Brelih 1980)

NN Mali Dolfin reef: 20.IX.1961 from *Podarcis sicula* (Tovornik and Brelih 1980)

NN, LL Lutrošnjak Island: 25.VII.1965 from *Podarcis sicula* (Tovornik and Brelih 1980)

N, LL Kamenjak Island: 25.VII.1965 from *Podarcis sicula* (Tovornik and Brelih 1980)

NN, LL Premuda Island: 1.VIII.1965 from *Podarcis sicula* (Tovornik and Brelih 1980)

LL Zapadni Greben Island: 23.VII.1965 from *Podarcis sicula* (Tovornik and Brelih 1980)

LL Južni Greben Island: 23.VII.1965 from *Podarcis sicula* (Tovornik and Brelih 1980)

NN, LL Silba Island: 13.VIII.1963 from *Podarcis sicula* (Tovornik and Brelih 1980)

NN, LL ibidem: 22.VII.1965 from *Podarcis sicula* (Tovornik and Brelih 1980)

LL ibidem: 2.VIII.1965 from *Podarcis sicula* (Tovornik and Brelih 1980)

NN, LL Morovnik Island: 24.VII.1965 from *Podarcis sicula* (Tovornik and Brelih 1980)

NN, LL Olib Island: 3.VIII.1965 from *Podarcis sicula* (Tovornik and Brelih 1980)

LL Planičić reef: 3.VIII.1965 from *Podarcis sicula* (Tovornik and Brelih 1980)

LL Šip reef: 24.VII.1965 from *Podarcis sicula* (Tovornik and Brelih 1980)

L Vela Sestrica Island: 11.VIII.1963 from *Podarcis sicula* (Tovornik and Brelih 1980)

N, LL Dužac Island: 11.VIII.1963 from *Podarcis sicula* (Tovornik and Brelih 1980)

NN, LL Golac Island: 9.VIII.1965 from *Podarcis sicula* (Tovornik and Brelih 1980)

NN Mali Laganj Island: 12.VIII.1963 from *Podarcis sicula* (Tovornik and Brelih 1980)

NN, LL Baričevac Island: 6.VIII.1965 from *Podarcis sicula* (Tovornik and Brelih 1980)

NN, LL Mežanj Island: 8.VIII.1965 from *Podarcis sicula* (Tovornik and Brelih 1980)

LL Kurjak reef: 24.VII. 1965 from *Podarcis melisellensis* (Tovornik and Brelih 1980)

NN, LL Sparušnjak Island: 7.VIII.1965 from *Podarcis melisellensis* (Tovornik and Brelih 1980)

NN, LL Maslinovac Island: 7.VIII.1963 from *Podarcis melisellensis* (Tovornik and Brelih 1980)

NN, LL Vrtlac Island: 8.VIII.1963 from *Podarcis melisellensis* (Tovornik and Brelih 1980)

LL Veseljuh Island: 28.VII.1964 from *Podarcis sicula* (Tovornik and Brelih 1980)

NN, LL Bisaga Island: 7.VIII.1964 from *Podarcis sicula* (Tovornik and Brelih 1980)

N, LL Mali Tetovišnjak Island: 22.VII.1964 from *Podarcis sicula* (Tovornik and Brelih 1980)

LL Čerigul Island: 22.VII.1964 from *Podarcis sicula* (Tovornik and Brelih 1980)

LL Mali Kamešnjak Island: 23.VII.1964 from *Podarcis sicula* (Tovornik and Brelih 1980)

NN, LL Samograd Island: 6.VIII.1963 from *Podarcis sicula* (Tovornik and Brelih 1980)

LL Koromašna Island: 20.VII.1964 from *Podarcis sicula* (Tovornik and Brelih 1980)

NN, LL Gušteranski Island: 23.VII.1964 from *Podarcis sicula* (Tovornik and Brelih 1980)

N, LL Palagruža Island: 8.VI.1960 from *Podarcis sicula* (Tovornik and Brelih 1980)

LL Kamik od Tramuntane Island: 9.VI.1960 from *Podarcis sicula* (Tovornik and Brelih 1980)

LL Brusnik Island: 7.VI.1960 from *Podarcis melisellensis* (Tovornik and Brelih 1980)

NN Pod Mrčaru reef: 31.VII.1971 from *Podarcis melisellensis* (Tovornik and Brelih 1980)

NN, LL Božikovac Island: 1.VIII.1964 from *Podarcis melisellensis* (Tovornik and Brelih 1980)

N, LL Mala Lavdara Island: 1.VIII.1964 from *Podarcis melisellensis* (Tovornik and Brelih 1980)

NN, LL Trimulići Island: 2.VIII.1964 from *Podarcis melisellensis* (Tovornik and Brelih 1980)

NN, LL Trstikovac Island: 2.VIII.1964 from *Podarcis melisellensis* (Tovornik and Brelih 1980)

LL Vela Skala Island: 2.VIII.1964 from *Podarcis melisellensis* (Tovornik and Brelih 1980)

NN, LL Mala Skala Island: 2.VIII.1964 from *Podarcis melisellensis* (Tovornik and Brelih 1980)

NN, LL Rončić Island: 1.VIII.1964 from *Podarcis melisellensis* (Tovornik and Brelih 1980)

NN, LL Mali Babuljaš Island: 7.VIII.1963 from *Podarcis melisellensis* (Tovornik and Brelih 1980)

NN, LL Malo Šilo Island: 6.VIII.1964 from *Podarcis melisellensis* (Tovornik and Brelih 1980)

NN, LL Mrtovac Island: 6.VIII.1964 from *Podarcis melisellensis* (Tovornik and Brelih 1980)

NN, LL Veli Babuljaš Island:7.VIII.1964 from *Podarcis melisellensis* (Tovornik and Brelih 1980)

NN, LL Mali Prišnjak Island: 7.VIII.1964 from *Podarcis melisellensis* (Tovornik and Brelih 1980)

NN, LL Vodenjak Island: 7.VIII.1964 from *Podarcis melisellensis* (Tovornik and Brelih 1980)

NN, LL Jančar Island: 7.VIII.1964 from *Podarcis melisellensis* (Tovornik and Brelih 1980)

NN, LL Purara Island: 7.VIII.1964 from *Podarcis melisellensis* (Tovornik and Brelih 1980)

NN, LL Mala Prduša Island: 7.VIII.1964 from *Podarcis melisellensis* (Tovornik and Brelih 1980)

NN, LL Babina Guzica Island: 8.VIII.1964 from *Podarcis melisellensis* (Tovornik and Brelih 1980)

LL Veli Puh Island: 21.VII.1964 from *Podarcis melisellensis* (Tovornik and Brelih 1980)

LL Vodeni Puh Island: 21.VII.1964 from *Podarcis melisellensis* (Tovornik and Brelih 1980)

NN, LL Kameni Puh Island: 21.VII.1964 from *Podarcis melisellensis* (Tovornik and Brelih 1980)

LL Čavlin Island: 22.VII.1964 from *Podarcis melisellensis* (Tovornik and Brelih 1980)

LL Veli Tetovišnjak Island: 22.VII.1964 from *Podarcis melisellensis* (Tovornik and Brelih 1980)

LL Veli Kamešnjak Island: 23.VII.1964 from *Podarcis melisellensis* (Tovornik and Brelih 1980)

N, LL Žirje Island: 27.VII.1964 from *Podarcis melisellensis* (Tovornik and Brelih 1980)

N, L Mikavica Island: 4.VIII.1964 from *Podarcis melisellensis* (Tovornik and Brelih 1980)

LL Škrovada reef: 23.VII.1964 from *Podarcis melisellensis* (Tovornik and Brelih 1980)

NN, LL Kosmerka Island: 4.VIII.1964 from *Podarcis melisellensis* (Tovornik and Brelih 1980)

L Pod Mrčaru reef: 31.VII.1971 from *Dalmatolacerta oxycephala* (Tovornik and Brelih 1980)

♂♂, ♀♀ Southern Croatia from *Ovis aries* and *Capra hircus* (Duh et al. 2006, 2010)

**7. *Haemaphysalis erinacei* Pavesi, 1884**

N Dubrovnik 10.VIII. 1964 from *Tachymarptis melba* (Tovornik 1990a)

**8. *Haemaphysalis parva* (Neumann, 1897)**

♂♂, ♀♀ Brač Island: (Tovornik 1980, Hubálek 2010)

**Genus** *Hyalomma* Koch, 1844

**9. *Hyalomma scupense* Schulze, 1919**

♂♂, ♀♀ Pula, months: III. IV. IX. 1936–1963 from *Ovis aries*, *Bos taurus* (Mikačić 1965)

♂♂, ♀♀ Krk Island, months: II. III. IV. 1936–1963 from *Ovis aries*, *Bos taurus* (Mikačić 1965)

♂♂, ♀♀ Lošinj Island: Veli Lošinj, month: IV. 1936–1963 from *Ovis aries*, *Bos taurus* (Mikačić 1965)

♂♂, ♀♀ Zadar, months: II. III. IV. VI. VIII. 1936–1963 from *Ovis aries*, *Bos taurus* (Mikačić 1965)

NN ibidem: months: IX. XI. 1936–1963 from *Ovis aries*, *Bos taurus* (Mikačić 1965)

♂♂, ♀♀ Split, months: I. II. III. IV. VI. 1936–1963 from *Ovis aries*, *Bos taurus* (Mikačić 1965)

NN ibidem: months: XI. XII. 1936–1963 from *Ovis aries*, *Bos taurus* (Mikačić 1965)

♂♂, ♀♀ Brač Island, months:V.VI. 1936–1963 from *Ovis aries*, *Bos taurus* (Mikačić 1965)

♂♂, ♀♀ ibidem: 1964/1965 from *Ovis aries* (Tovornik 1976b)

♂♂, ♀♀ ibidem: -, (Hubálek 2010)

♂♂, ♀♀, NN Dubrovnik, month: I. 1936–1963 from *Ovis aries*, *Bos taurus* (Mikačić 1965)

♂♂, ♀♀ ibidem: months: II. III. IV.V. IX. XI. XII.1936–1963 from *Ovis aries*, *Bos taurus* (Mikačić 1965)

**10. *Hyalomma marginatum* Koch, 1844**

♂♂, ♀♀ Pula, months: I. II. III. IV. V. VI. VII. VIII. IX. X. XI. XII. 1936–1963 from *Ovis aries*, *Equus caballus*, *Bos taurus* (Mikačić 1965)

♂♂, ♀♀ Krk Island, months: II. III. IV. V. VI. VII. VIII. IX. X. 1936–1963 from *Ovis aries*, *Equus caballus*, *Bos taurus* (Mikačić 1965)

♂♂, ♀♀ Cres Island, months: IV. VI. VII. X. XII. 1936–1963 from *Ovis aries*, *Equus caballus*, *Bos taurus* (Mikačić 1965)

♂♂, ♀♀ Lošinj Island: Veli Lošinj, months: II. III. IV. V. VI. VII. 1936–1963 from *Ovis aries*, *Equus caballus*, *Bos taurus* (Mikačić 1965)

♂♂, ♀♀ Zadar, months: II. III. IV. V. VI. VII. VIII. X. 1936–1963 from *Ovis aries*, *Equus caballus*, *Bos taurus* (Mikačić 1965)

♂♂, ♀♀ Split, months: II. III. IV. V. VI. VII. VIII. IX. X. XII. 1936–1963 from *Ovis aries*, *Equus caballus*, *Bos taurus* (Mikačić 1965)

♂♂, ♀♀ Dubrovnik, months: III. IV. V. VI. VII. VIII. IX. XII. 1936–1963 from *Ovis aries*, *Equus caballus*, *Bos taurus* (Mikačić 1965)

♂, ♀ Petrinja, month: V. from *Equus caballus*, *Bos taurus* (Mikačić 1968)

♂♂, ♀♀ Brač Island, months: I. II. III. IV. V. VI. VII. VIII. IX. X. 1936–1963 from *Ovis aries*, *Equus caballus*, *Bos taurus* (Mikačić 1965)

♂♂, ♀♀ ibidem: months: V. VI. VII. VIII. IX. X. 1964/1965 from *Ovis aries* (Tovornik 1976b)

♂, ♀ ibidem: Blato, month: IV.1964/1965 from *Ovis aries* (Tovornik 1976b)

♂♂, ♀♀ ibidem: Zapadna Laščatna, months: IV. VII.1964/1965 collected on vegetation and/or the ground by flagging/dragging method (Tovornik 1976b)

♂♂, ♀♀ ibidem: (Hubálek 2010)

NN, LL ibidem: month: VII. 1967 from *Erinaceus europaeus* (Tovornik 1987b)

♂♂, ♀♀ Kaštela Bay, month: X. 2000 from *Ovis aries*, *Capra hircus*, *Bos taurus* (Punda -Polić et al. 2002)

♂♂, ♀♀ Sinj, month: X. 2000 from *Ovis aries*, *Capra hircus*, *Bos taurus* (Punda-Polić et al. 2002)

**Genus** *Rhipicephalus* Koch, 1844

**11. *Rhipicephalus annulatus* (Say, 1821)**

♂♂, ♀♀ Lošinj Island: Veli Lošinj 29.IX.–1.X.1948 from *Bos taurus* (Mikačić 1949)

♂♂, ♀♀ ibidem: months: IV. V. X. 1936–1963 from *Equus caballus*, *Bos taurus* (Mikačić 1965)

♂♂, ♀♀ Zadar, months: III. IV.V. VI. VII. VIII. IX. 1936–1963 from *Equus caballus*, *Bos taurus* (Mikačić 1965)

NN ibidem: months: IV. VII. VIII. IX. 1936–1963 from *Equus caballus*, *Bos taurus* (Mikačić 1965)

♂♂, ♀♀ Split, months: VI. VII. VIII. IX. X. XI. 1936–1963 from *Equus caballus*, *Bos taurus* (Mikačić 1965)

NN ibidem: months: IX. XI. 1936–1963 from *Equus caballus*, *Bos taurus* (Mikačić 1965)

♂♂, ♀♀ Brač Island: month: VII. 1936–1963 from *Equus caballus*, *Bos taurus* (Mikačić 1965)

♂♂, ♀♀ ibidem: (Hubálek 2010)

♂♂, ♀♀ Dubrovnik, months: III. IV. V. VII. 1936–1963 from *Equus caballus*, *Bos taurus* (Mikačić 1965)

NN ibidem: months: IV.XI. 1936–1963 from *Equus caballus*, *Bos taurus* (Mikačić 1965)

**12. *Rhipicephalus bursa* Canestrini & Fanzago, 1878**

♂♂, ♀♀ Lošinj Island: Čunski-Like 6.–11.IV.1948 from *Ovis aries* (Mikačić 1949)

♂♂, ♀♀ Cres Island, months:, IV. V. 1948 from *Ovis aries* (Mikačić 1949)

♂♂, ♀♀ ibidem: month: VIII. 1948 from *Ovis aries* (Mikačić 1949)

LL ibidem: 29.IX.–1.X.1948 from *Ovis aries* (Mikačić 1949)

♂♂, ♀♀, NN, LL ibidem: Hrasta 6.–11.IV. 1948 from *Ovis aries* (Mikačić 1949)

♂♂, ♀♀, NN, LL ibidem: 29.IX.–1.X.1948 from *Ovis aries* (Mikačić 1949)

NN, L ibidem: 10.XI.1948 from *Ovis aries* (Mikačić 1949)

NN ibidem: Vrana 6.–11.IV. 1948 from *Capra hircus* (Mikačić 1949)

♂♂, ♀♀, NN ibidem: Vodice 6.–11.IV. 1948 from *Ovis aries* (Mikačić 1949)

♂♂, ♀♀ ibidem: Orlec 6.–11.IV. 1948 from *Ovis aries* (Mikačić 1949)

♂♂, ♀♀ ibidem: Belej 29.IX.–1.X.1948 from *Ovis aries* (Mikačić 1949)

NN ibidem: 4.XI.1948 from *Ovis aries* (Mikačić 1949)

♂♂, ♀♀ Pula, months: II. III. IV. V. VI. VIII. 1936–1963 from *Ovis aries*, *Equus caballus*, *Bos taurus*, *Capra hircus* (Mikačić 1965)

NN ibidem: months: I. II. III. IV. X. XI. XII. 1936–1963 from *Ovis aries*, *Equus caballus*, *Bos taurus*, *Capra hircus* (Mikačić 1965)

LL ibidem: months: III. IV. XI. 1936–1963 from *Ovis aries*, *Equus caballus*, *Bos taurus*, *Capra hircus* (Mikačić 1965)

♂♂, ♀♀ Krk Island, months: II. III. IV. V. VI. VII.VIII. X. 1936–1963 from *Ovis aries*, *Equus caballus*, *Bos taurus*, *Capra hircus* (Mikačić 1965)

NN ibidem: months: I. III. IV.VI. X. XI. XII.1936–1963 from *Ovis aries*, *Equus caballus*, *Bos taurus*, *Capra hircus* (Mikačić 1965)

LL ibidem: months: III. IV. X. 1936–1963 from *Ovis aries*, *Equus caballus*, *Bos taurus*, *Capra hircus* (Mikačić 1965)

♂♂, ♀♀ Cres Island: Cres, months: III. IV. V. VI. VII. IX. 1936–1963 from *Ovis aries*, *Equus caballus*, *Bos taurus*, *Capra hircus* (Mikačić 1965)

NN ibidem: months: I. II. III. IV. V. XI. 1936–1963 from *Ovis aries*, *Equus caballus*, *Bos taurus*, *Capra hircus* (Mikačić 1965)

LL ibidem: months: I. III. XI. 1936–1963 from *Ovis aries*, *Equus caballus*, *Bos taurus*, *Capra hircus* (Mikačić 1965)

♂♂, ♀♀ Lošinj Island: Veli Lošinj, months: III. IV. V. VI. VII. VIII. IX. 1936–1963 from *Ovis aries*, *Equus caballus*, *Bos taurus*, *Capra hircus* (Mikačić 1965)

NN ibidem: months: II. III. IV.X. XI. XII. 1936–1963 from *Ovis aries*, *Equus caballus*, *Bos taurus*, *Capra hircus* (Mikačić 1965)

LL ibidem: months: III. IV. IX. XI. 1936–1963 from *Ovis aries*, *Equus caballus*, *Bos taurus*, *Capra hircus* (Mikačić 1965)

♂♂, ♀♀ Zadar, months: III. IV. V. VI.VII. VIII. 1936–1963 from *Ovis aries*, *Equus caballus*, *Bos taurus*, *Capra hircus* (Mikačić 1965)

NN ibidem: months: I. II. III. IV. V. XII. 1936–1963 from *Ovis aries*, *Equus caballus*, *Bos taurus*, *Capra hircus* (Mikačić 1965)

LL ibidem: month: I. III. IV. 1936–1963 from *Ovis aries*, *Equus caballus*, *Bos taurus*, *Capra hircus* (Mikačić 1965)

♂♂, ♀♀ Split, months: II. III. V. VI. VII. VIII. IX. 1936–1963 from *Ovis aries*, *Equus caballus*, *Bos taurus*, *Capra hircus* (Mikačić 1965)

NN ibidem: months; II. III. X. XII. 1936–1963 from *Ovis aries*, *Equus caballus*, *Bos taurus*, *Capra hircus* (Mikačić 1965)

LL ibidem: month: III. 1936–1963 from *Ovis aries*, *Equus caballus*, *Bos taurus*, *Capra hircus* (Mikačić 1965)

♂♂, ♀♀ Brač Island, months: II. III. IV. V.VI. VII.VIII. 1936–1963 from *Ovis aries*, *Equus caballus*, *Bos taurus*, *Capra hircus* (Mikačić 1965)

NN ibidem: months: I. II. III. IV. X. XI. XII. 1936–1963 from *Ovis aries*, *Equus caballus*, *Bos taurus*, *Capra hircus* (Mikačić 1965)

♂♂, ♀♀ ibidem: 1964/1965 from *Ovis aries* (Tovornik 1976b)

♂♂, ♀♀ ibidem: (Hubálek 2010)

NN ibidem: Blato 4.–9.XI. 1965 from *Ovis aries* (Tovornik and Černy 1972)

♂, ♀ ibidem: Blato, month: IX.1964/1965 collected on vegetation and/or the ground by flagging/dragging method (Tovornik 1976b)

♂♂, ♀♀ ibidem: Sveti Toma, months: IX. X. 1964/1965 collected on vegetation and/or the ground by flagging/dragging method (Tovornik 1976b)

♂♂, ♀♀ ibidem: Zapadna Laščatna, months: VII. VIII. 1964/1965 collected on vegetation and/or the ground by flagging/dragging method (Tovornik 1976b)

♂♂, ♀♀ Dubrovnik, months: III. IV.V. VI.VII. VIII. IX. 1936–1963 from *Ovis aries*, *Equus caballus*, *Bos taurus*, *Capra hircus* (Mikačić 1965)

NN ibidem: months: II. IV. V. X. XI. XII. 1936–1963 from *Ovis aries*, *Equus caballus*, *Bos taurus*, *Capra hircus* (Mikačić 1965)

♂♂, ♀♀ Kaštela Bay, month: X. 2000 from *Ovis aries*, *Capra hircus*, *Bos taurus* (Punda-Polić et al. 2002)

♂♂, ♀♀ Sinj, month: X. 2000 from *Ovis aries*, *Capra hircus*, *Bos taurus* (Punda-Polić et al. 2002)

**13. *Rhipicephalus sanguineus* (Latreille, 1806)**

♂♂, ♀♀ Mali Lošinj Island: Čunski-Like 6.–11.IV. 1948 -, V. 1948 from *Ovis aries* (Mikačić 1949)

♂♂, ♀♀ Cres Island: Hrasta 6.–11.IV. 1948 from *Ovis aries* (Mikačić 1949)

♂♂, ♀♀ ibidem: Belej 6.–11.IV. 1948 from *Ovis aries* (Mikačić 1949)

♂♂, ♀♀ ibidem: Vrana 6.–11.IV. 1948 from *Capra hircus* (Mikačić 1949)

♂♂, ♀♀ ibidem: Vodice 6.–11.IV. 1948 from *Ovis aries* (Mikačić 1949)

♂♂, ♀♀ ibidem: Orlec. months: IV. V. 1948 from *Ovis aries* (Mikačić 1949)

♂♂, ♀♀ ibidem: Cres, months: IV. V. VII. 1936–1963 from *Ovis aries*, *Bos taurus*, *Canis lupus familiaris* (Mikačić 1965)

♂♂, ♀♀ Pula, months: V.VI. 1936–1963 from *Ovis aries*, *Bos taurus*, *Canis lupus familiaris* (Mikačić 1965)

♂ ibidem: 18.V.1973 from *Canis lupus familiaris* (Tovornik and Vesenjak-Hirjan 1988)

♂♂, ♀♀ Zadar, months: III. IV. V. VI. 1936–1963 from *Ovis aries*, *Bos taurus*, *Canis lupus familiaris* (Mikačić 1965)

♂♂, ♀♀ Split, months: III. IV. V. VI. 1936–1963 from *Ovis aries*, *Bos taurus*, *Canis lupus familiaris* (Mikačić 1965)

♂♂, ♀♀ Dubrovnik, months: III. IV. V. VI. VII. VIII. 1936–1963 from *Ovis aries*, *Bos taurus*, *Canis lupus familiaris* (Mikačić 1965)

♂♂, ♀♀ Brač Island, months: II. III. IV. 1936–1963 from *Ovis aries*, *Bos taurus*, *Canis lupus familiaris* (Mikačić 1965)

♂♂, ♀♀ ibidem: from 1964/1965 from *Ovis aries* (Tovornik 1976b)

♂♂, ♀♀ ibidem: Blato, months; IV. V. VI. 1964/1965 collected on vegetation and/or the ground by dragging/flagging method (Tovornik 1976b)

♂♂, ♀♀ ibidem: Sveti Toma, months: IV. V. VI. VII. 1964/1965 collected on vegetation and/or the ground by dragging/flagging method (Tovornik 1976b)

♂♂, ♀♀ ibidem: Zapadna Laščatna, months: IV. VI. VII.1964/1965 collected on vegetation and/or the ground by dragging/flagging method (Tovornik 1976b)

♂♂, ♀♀ ibidem: Sjeverna Laščatna, months: IV. VI. 1964/1965 collected on vegetation and/or the ground by dragging/flagging method (Tovornik 1976b)

♂♂, ♀♀ ibidem: 1.–4.V. 1964 and 28.III.–1.IV.1965 collected on vegetation by dragging method (Tovornik and Vesenjak-Hirjan 1988)

♂♂, ♀♀ ibidem: 28.III. , 27.IV., 1.VI., 4.VI. 1965 from *Ovis aries*, (Tovornik and Vesenjak -Hirjan 1988)

♂♂, ♀♀ ibidem: (Hubálek 2010)

♂♂, ♀♀ Umag 27.V.1978 from *Canis lupus familiaris* (Tovornik 1987b)

♀ Slano (Dubrovnik) 10.V.1953 from *Canis aureus* (Tovornik and Vesenjak -Hirjan 1988)

♂♂, ♀♀ Ugljan Island: Kali 9.–20.V.1955 from *Ovis aries* (Tovornik and Vesenjak -Hirjan 1988)

♂♂, ♀♀ Rovanjska 23.IV.1958 from *Ovis aries* (Tovornik and Vesenjak-Hirjan 1988)

♂♂, ♀♀ Zadar 25.IV.1958 from *Ovis aries* (Tovornik and Vesenjak-Hirjan 1988)

♂♂ Pašman Island: 2.V.1985 from *Ovis aries* (Tovornik and Vesenjak-Hirjan 1988)

♂,♀ Dugi otok Island: 29.IV.1986 from *Ovis aries* (Tovornik and Vesenjak-Hirjan 1988)

♂♂, ♀♀ Mljet Island: Babino polje 8.–9.IV.1981, 16.–17.V. 1981 from *Capra hircus*, *Ovis aries*, *Canis lupus familiaris*, *Equus asinus* (Tovornik and Vesenjak -Hirjan 1988)

♂♂, ♀♀ ibidem: Zadublje 8.–9.IV.1981 from *Capra hircus*, *Ovis aries*, *Canis lupus familiaris*, *Equus asinus* (Tovornik and Vesenjak-Hirjan 1989)

♂♂, ♀♀ ibidem: Veliko jezero 8.–9.IV.1981 from *Capra hircus*, *Ovis aries*, *Canis lupus familiaris*, *Equus asinus* (Tovornik and Vesenjak-Hirjan 1989)

♂♂, ♀♀ ibidem: Soline 8.–9.IV.1981 from *Capra hircus*, *Ovis aries*, *Canis lupus familiaris*, *Equus asinus* (Tovornik and Vesenjak-Hirjan 1989)

♂♂, ♀♀ ibidem: Babino polje 16.–17.V. 1981 from *Capra hircus*, *Ovis aries*, *Canis lupus familiaris*, *Equus asinus* (Tovornik and Vesenjak-Hirjan 1989)

♂♂, ♀♀ ibidem: Blato 16.–17.V. 1981 from *Capra hircus*, *Ovis aries*, *Canis lupus familiaris, Equus asinus* (Tovornik and Vesenjak-Hirjan 1989)

**14. *Rhipicephalus turanicus* Pomerantsev, 1940**

♂♂, ♀♀ Ugljan Island: Kali 9.–20.V.1955 from *Ovis aries* (Tovornik and Vesenjak -Hirjan 1988)

♂ Zadar 25.IV. 1958 from *Ovis aries* (Tovornik and Vesenjak-Hirjan 1988)

♂ Lokrum Island: 9.IX.1963 from *Capreolus capreolus* (Tovornik and Vesenjak-Hirjan 1988)

♂ ibidem: 4.IX. 1963 from *Capreolus capreolus* (Tovornik 1988a)

♂♂, ♀ Brač Island: 1.–4.V.1964 and 28.III.–1.IV.1965 collected on vegetation by dragging method (Tovornik and Vesenjak-Hirjan 1988)

♂♂, ♀♀ ibidem: 28.III., 27.IV., 1.VI., 4.VI., 1965 from *Ovis aries* (Tovornik and Vesenjak-Hirjan 1988)

♂♂, ♀♀ Pašman Island: 2.V.1985 from *Ovis aries* (Tovornik and Vesenjak-Hirjan 1988)

♂♂, ♀ Dugi otok Island: 29.IV.1986 from *Ovis aries* (Tovornik and Vesenjak -Hirjan 1988)

♂♂, ♀♀ Mljet Island: 8.–9.IV. 1981, 16–17.V. 1981 from *Capra hircus* and other domestic animals (Tovornik and Vesenjak-Hirjan 1988)

♂♂, ♀♀ ibidem: Babino polje 8.–9.IV.1981 from *Capra hircus*, *Ovis aries*, *Felis catus*, *Canis lupus familiaris* (Tovornik and Vesenjak-Hirjan 1989)

♂♂, ♀♀ ibidem: Zadublje 8.–9.IV.1981 from *Capra hircus*, *Ovis aries*, *Canis lupus familiaris*, *Felis catus* (Tovornik and Vesenjak-Hirjan 1989)

♂♂, ♀♀ ibidem: Veliko jezero 8.–9.IV.1981 from *Capra hircus*, *Ovis aries*, *Canis lupus familiaris*, *Felis catus* (Tovornik and Vesenjak-Hirjan 1989)

♂♂, ♀♀ ibidem: Soline 8.–9.IV.1981 from *Capra hircus*, *Ovis aries*, *Canis lupus familiaris*, *Felis catus* (Tovornik and Vesenjak-Hirjan 1989)

♂♂, ♀♀ ibidem: Babino polje 16.–17.V. 1981 from *Capra hircus*, *Ovis aries*, *Canis lupus familiaris*, *Felis catus* (Tovornik and Vesenjak-Hirjan 1989)

♂♂, ♀♀ ibidem: Blato 16.–17.V. 1981 from *Capra hircus*, *Ovis aries*, *Canis lupus familiaris,Felis catus* (Tovornik and Vesenjak-Hirjan 1989)

**Genus** *Ixodes* Latreille, 1795

**15. *Ixodes arboricola* Schulze & Schlottke, 1930**

♀♀ Zagreb 29.IX. 1962 from *Turdus merula* (Tovornik 1990a, Tovornik 1991b)

**16. *Ixodes frontalis* (Panzer, 1798)**

♀ Velebit mountain: Donja Klada, 1975 from *Martes foina* (Tovornik 1987b)

♀ Dubrovnik 22.II.1962 from *Bombycilla garrulus* (Tovornik 1990a, Tovornik 1991b)

♀ ibidem: 1.XII. 1967 from *Phoenicurus ochruros* (Tovornik 1990a, Tovornik 1991b)

♀ Zagreb, vicinity, 20.IV. 1975 from *Martes foina* (Tovornik 1991b)

**17. *Ixodes gibbosus* Nuttal, 1916**

♂♂, ♀♀ Brač Island: Sveti Toma 4–9. XI. 1965 from *Ovis aries* (Tovornik and Černy 1972)

♂♂, ♀♀ ibidem: Osridke 4–9.XI.1965 from *Ovis aries* (Tovornik and Černy 1972)

♂♂, ♀♀ ibidem: Blato 4.–9.XI. 1965 from *Ovis aries* (Tovornik and Černy 1972)

♂♂, ♀♀, NN, LL ibidem: Zapadna Laščatna 24.–25.XI. 1964 collected on the vegetation by dragging method (Tovornik and Černy 1972)

♂♂, ♀♀ ibidem: Blato, months: I. II. IV. V. XII. 1964/1965 collected on vegetation and/or the ground by dragging/flagging method (Tovornik 1976b)

♂♂, ♀♀ ibidem: Sveti Toma, months: I. II. IV. V. XI. XII. 1964/1965 collected on vegetation and/or the ground by dragging/flagging method (Tovornik 1976b)

♂♂, ♀♀ ibidem: Zapadna Laščatna, months: I. IV. XI. XII. 1964/1965 collected on vegetation and/or the ground by dragging/flagging method (Tovornik 1976b)

♂♂, ♀♀ ibidem: Sjeverna Laščatna, months: I. IV. XI. XII. 1964/1965 collected on vegetation and/or the ground by dragging/flagging method (Tovornik 1976b, Tovornik and Šooš 1976)

♂♂, ♀♀ ibidem: Supetar 22.II. 1974 from *Ovis aries* (Tovornik 1991a)

♂♂, ♀♀ ibidem: (Hubálek 2010)

**18. *Ixodes hexagonus* Leach, 1815**

♂♂, ♀♀ Gornja Posavina, month: III. from *Equus caballus*, *Bos taurus* (Mikačić 1968)

NN Velika Kapela mountain: Razvala 16.VIII. 1969 from *Erinaceus concolor* (Tovornik 1987b)

♀ Dugo Selo 5.IV. 1959 from *Martes martes* (Tovornik 1987b)

♀♀ Đakovo 7.IV.–25.IV. 2011 from *Canis lupus familiaris*

♂♂ ibidem: 2.V.–25.V. 2011 from *Canis lupus familiaris*

**19. *Ixodes ricinus* (Linnaeus, 1758)**

♂♂, ♀♀, NN, LL Cres Island: Hrasta 6.–11.IV.1948 from *Ovis aries* (Mikačić 1949)

♂, ♀♀ ibidem: 10.XI.1948 from *Ovis aries* (Mikačić 1949)

♂♂, ♀♀, NN ibidem: Vrana 6.–11.IV.1948 from *Capra hircus* (Mikačić 1949)

♂♂, ♀♀, NN, LL ibidem: Vodice 6.–11.IV.1948 from *Ovis aries* (Mikačić 1949)

NN ibidem: Cres 6.–11.IV.1948 from *Capra hircus* (Mikačić 1949)

♂♂, ♀♀, NN ibidem: Belej 4.XI.1948 from *Ovis aries* (Mikačić 1949)

♂♂, ♀♀ Pula, months: I. II. III. IV. V. VI. VIII. IX. X. XI. XII. 1936–1963 from *Ovis aries*, *Bos taurus*, *Capra hircus*, *Equus caballus*, *Equus asinus* (Mikačić 1965)

NN ibidem, months: I. II. III. IV. X. XII. 1936–1963 from *Ovis aries*, *Bos taurus*, *Capra hircus*, *Equus caballus*, *Equus asinus* (Mikačić 1965)

LL ibidem, months: IV. VII. XII. 1936–1963 from *Ovis aries*, *Bos taurus*, *Capra hircus*, *Equus caballus*, *Equus asinus* (Mikačić 1965)

♂♂, ♀♀ Krk Island, months: I. II. III. IV. V. IX. XI. XII. 1936–1963 from *Ovis aries*, *Bos taurus*, *Capra hircus*, *Equus caballus*, *Equus asinus* (Mikačić 1965)

NN ibidem, months: I. II. III. IV. IX. X. 1936–1963 from *Ovis aries*, *Bos taurus*, *Capra hircus*, *Equus caballus*, *Equus asinus* (Mikačić 1965)

LL ibidem, months: I. II. III. IV. X. 1936–1963 from *Ovis aries*, *Bos taurus*, *Capra hircus*, *Equus caballus*, *Equus asinus* (Mikačić 1965)

♂♂, ♀♀ Cres Island, months: I. II. III. IV.V. IX. X. XI. XII. 1936–1963 from *Ovis aries*, *Bos taurus*, *Capra hircus*, *Equus caballus*, *Equus asinus* (Mikačić 1965)

NN ibidem, months: I. II. III. IV. IX. XI. 1936–1963 from *Ovis aries*, *Bos taurus*, *Capra hircus*, *Equus caballus*, *Equus asinus* (Mikačić 1965)

LL ibidem, months: II. III. 1936–1963 from *Ovis aries*, *Bos taurus*, *Capra hircus*, *Equus caballus*, *Equus asinus* (Mikačić 1965)

♂♂, ♀♀ Lošinj Island: Veli Lošinj, months: I. II. III. IV. V. IX. X. XI. XII. 1936–1963 from *Ovis aries*, *Bos taurus*, *Capra hircus*, *Equus caballus*, *Equus asinus* (Mikačić 1965)

NN ibidem: months: I. II. III. IV. V. IX. X. XI. 1936–1963 from *Ovis aries*, *Bos taurus*, *Capra hircus*, *Equus caballus*, *Equus asinus* (Mikačić 1965)

LL ibidem: months: II. III. IV. X. 1936–1963 from *Ovis aries*, *Bos taurus*, *Capra hircus*, *Equus caballus*, *Equus asinus* (Mikačić 1965)

♂♂, ♀♀ Zadar, months: I. II. III. IV. VI. IX. X. XI. XII. 1936–1963 from *Ovis aries*, *Bos taurus*, *Capra hircus*, *Equus caballus*, *Equus asinus* (Mikačić 1965)

NN ibidem: months: I. III. IV. IX. 1936–1963 from *Ovis aries*, *Bos taurus*, *Capra hircus*, *Equus caballus*, *Equus asinus* (Mikačić 1965)

LL ibidem: months: I. IV. IX. 1936–1963 from *Ovis aries*, *Bos taurus*, *Capra hircus*, *Equus caballus*, *Equus asinus* (Mikačić 1965)

♂♂, ♀♀ Split, months: I. II. III. IV. X. XI. XII. 1936–1963 from *Ovis aries*, *Bos taurus*, *Capra hircus*, *Equus caballus*, *Equus asinus* (Mikačić 1965)

NN ibidem: months: I. II. III.1936–1963 from *Ovis aries*, *Bos taurus*,*Capra hircus*, *Equus caballus*, *Equus asinus* (Mikačić 1965)

LL ibidem: month: II. 1936–1963 from *Ovis aries*, *Bos taurus*, *Capra hircus*, *Equus caballus*, *Equus asinus* (Mikačić 1965)

♂♂, ♀♀ Brač Island, months: I. III. IX. X. XI. XII. 1936–1963 from *Ovis aries*, *Bos taurus*, *Capra hircus*, *Equus caballus*, *Equus asinus* (Mikačić 1965)

NN ibidem: months: I. II. III. 1936–1963 from *Ovis aries*, *Bos taurus*, *Capra hircus*, *Equus caballus*, *Equus asinus* (Mikačić 1965)

♂♂, ♀♀ Dubrovnik, months: I. II. III. IV.V. IX. X. XI. XII. 1936–1963 from *Ovis aries*, *Bos taurus*, *Capra hircus*, *Equus caballus*, *Equus asinus* (Mikačić 1965)

NN ibidem: months: I. II. IV. XI. XII.1936–1963 from *Ovis aries*, *Bos taurus*, *Capra hircus*, *Equus caballus*, *Equus asinus* (Mikačić 1965)

N, L Rovinj 7.IV.1959 from *Algyroides nigropunctatus* (Tovornik and Brelih 1980)

N, L ibidem: 7.IV.1959 from *Podarcis melisellensis* (Tovornik and Brelih 1980)

L ibidem: 10.IV. 1959 from *Podarcis melisellensis* (Tovornik and Brelih 1980)

NN, LL Tovarjež Island: 7.V.1959 from *Podarcis sicula* (Tovornik and Brelih 1980)

N, L Lakal Island: 7.V.1959 from *Podarcis sicula* (Tovornik and Brelih 1980)

NN, LL Lunga Island: 6.IV.1959 from *Podarcis sicula* (Tovornik and Brelih 1980)

NN, LL Koversada Island: 4.IV.1959 from *Podarcis sicula* (Tovornik and Brelih 1980)

N, L Figarola Island: 9.IV.1959 from *Podarcis sicula* (Tovornik and Brelih 1980)

N, L Banjol Island: 11.IV.1959 from *Podarcis sicula* (Tovornik and Brelih 1980)

N Veliki Piruzi reef: 4.IV.1959 from *Podarcis sicula* (Tovornik and Brelih 1980)

LL Sturag Island: 12.VIII.1959 from *Podarcis sicula* (Tovornik and Brelih 1980)

LL Sveti Ivan Island: 12.VIII.1959 from *Podarcis sicula* (Tovornik and Brelih 1980)

L Sveti Ivan na Pučini reef: 4.IV.1959 from *Podarcis sicula* (Tovornik and Brelih 1980)

NN, LL Pulari Island: 8.V.1959 from *Podarcis sicula* (Tovornik and Brelih 1980)

LL Revera Island: 8.V.1959 from *Podarcis sicula* (Tovornik and Brelih 1980)

N, LL Veštar Island: 8.IV.1959 from *Podarcis sicula* (Tovornik and Brelih 1980)

NN, LL Velika Sestrica Island: 8.IV.1959 from *Podarcis sicula* (Tovornik and Brelih 1980)

NN, LL Pisulj Island: 8.V.1959 from *Podarcis sicula* (Tovornik and Brelih 1980)

NN, LL Gustinja Island: 8.V.1959 from *Podarcis sicula* (Tovornik and Brelih 1980)

NN, LL Unije Island: 17.VII.1960 from *Podarcis melisellensis*(Tovornik and Brelih 1980)

NN, LL ibidem: 17.VII. 1960 from *Podarcis sicula* (Tovornik and Brelih 1980)

N, LL Zeča Island: 18.VIII.1963 from *Podarcis sicula* (Tovornik and Brelih 1980)

N, LL Vele Srakane Island: 19.VII.1960 from *Podarcis sicula* (Tovornik and Brelih 1980)

LL Susak Island: 19.VII.1960 from *Podarcis sicula* (Tovornik and Brelih 1980)

NN, LL Oruda Island: 19.VII.1960 from *Podarcis sicula* (Tovornik and Brelih 1980)

NN, LL Palacol Island: 19.VII.1960 from *Podarcis sicula* (Tovornik and Brelih 1980)

N, LL Male Orjule Island: 19.VII.1960 from *Podarcis sicula* (Tovornik and Brelih 1980)

N, LL Školjić Island: 19.VII.1960 from *Podarcis sicula* (Tovornik and Brelih 1980)

NN, LL Sveti Petar Island: 29.VII.1965 from *Podarcis sicula* (Tovornik and Brelih 1980)

LL Ilovik Island: 29.VII.1965 from *Podarcis sicula* (Tovornik and Brelih 1980)

LL Grujica Island: 28.VII.1965 from *Podarcis sicula* (Tovornik and Brelih 1980)

♂♂, ♀♀, NN, LL Srednja Posavina: Stružec, 1970–1978 collected on vegetation and/or the ground by dragging/flagging method (Borčić et al. 1978)

♂♂, ♀♀ Gornja Posavina, months: III. IV. V. VI. VII. IX. X. XI. (Mikačić 1968)

NN ibidem, months: III. IV. V. VI. (Mikačić 1968)

LL ibidem, months: IV. V. (Mikačić 1968)

♂♂, ♀♀ Stara Ves 22.III.1963 collected in the forest biotopes (Tovornik 1976a, Vesenjak-Hirjan 1976)

♀ Zagreb 5.IV. 1959 from *Martes martes* (Tovornik 1987b)

LL Velika Kapela mountain: Razvala 16.VIII.1968 from *Clethrionomys glareolus* (Tovornik 1988b)

N, LL Lika: Plitvice, Crna rijeka 12.VIII. 1968 from *Apodemus sylvaticus* (Tovornik 1988b)

N, LL ibidem: 12.VIII. 1968 from *Apodemus flavicollis* (Tovornik 1988b)

N Vukomeričke gorice: Prkovec 19.VII. 1974 from *Apodemus flavicollis* (Tovornik 1988b)

L Kozjak mountain: Malačka 29.IV. 1974 from *Apodemus mystacinus* (Tovornik 1988b)

♀♀, N Dugo Selo 9.X.1964 from *Capreolus capreolus* (Tovornik 1988a)

♂, ♀♀, NN Dekanovec from *Capreolus capreolus* (Tovornik 1988a)

♂♂, ♀♀, NN Nedelišće 3.V.1978 from *Capreolus capreolus* (Tovornik 1988a)

N Dubrovnik 12.II. 1966 from *Buteo buteo* (Tovornik 1990a)

♀, NN, LL Stara Ves 21.IV. 1964 from *Anthus trivialis* (Tovornik 1990a)

NN, LL ibidem: 10.V. 1964 from *Anthus trivialis* (Tovornik 1990a)

NN, LL ibidem: 24.VI.1964 from *Pica pica* (Tovornik 1990a)

 ibidem: 15.,16. VII. 1964 from *Pica pica* (Tovornik 1990a)

N Baćinska jezera 30.IV. 1976 from *Cettia cetti* (Tovornik 1990a)

L Vrana-Biograd 25.IV. 1974 from *Luscinia megarhynchos* (Tovornik 1990a)

NN ibidem: 25.IV.1974 from *Turdus merula* (Tovornik 1990a)

NN Metković-Gabela 14.X. 1964 from *Turdus philomelos* (Tovornik 1990a)

L Peščenica-Vratovo 26–27.IV.1984 from *Clethrionomys glareolus* (Tovornik and Matjašič 1991)

N ibidem: month: V. 1984 from *Apodemus flavicollis* (Tovornik and Matjašič 1991)

N, LL ibidem: 3.VI. 1984 from *Apodemus flavicollis* (Tovornik and Matjašič 1991)

NN ibidem: 1.VII.1984 from *Apodemus flavicollis* (Tovornik and Matjašič 1991)

LL ibidem: 2.VII.1984 from *Apodemus flavicollis* (Tovornik and Matjašič 1991)

N Peščenica-Vratovo 22.–23.III.1984 from *Apodemus agrarius* (Tovornik and Matjašič 1991)

N ibidem: 2.VII.1984 from *Clethrionomys glareolus* (Tovornik and Matjašič 1991)

♂♂, ♀♀ Northern Croatia (Rijpkema et al. 1996)

♂♂, ♀♀ Lukovdol, Lipov vrh months: V.- VIII. 2008 from *Capreolus capreolus* (Pintur et al. 2012)

♂♂, ♀♀, NN Zmajevac 5.III.–30.III. 2011 from *Felis catus*

♂♂, ♀♀, NN ibidem: 10. IV.–24.IV. 2011 from *Felis catus*

♂♂, ♀♀, NN ibidem: 25.IV.- 30.IV. 2011 from *Felis catus*

♂♂, ♀♀, N ibidem: 7.V.- 25.V. 2011 from *Felis catus*

LL ibidem: 31.VII.2011 from *Homo sapiens*

♀ ibidem: 26.X. 2011 from *Felis catus*

♂, ♀♀, NN Đakovo 10.III.–30.III. 2011 from *Canis lupus familiaris*

♂♂, ♀♀, NN ibidem: 7.IV.- 25.IV. 2011 from *Canis lupus familiaris*

♀♀ ibidem: 2.V.–25.V. 2011 from *Canis lupus familiaris*

♀, NN Sungerski Lug 25.VI. 2011 from *Canis lupus familiaris*

♂♂, ♀♀, NN Mikleuš 27.IV. 2011 collected on vegetation using the cloth-dragging method

♂♂, ♀♀, NN, L ibidem: 14.V. 2011 collected on vegetation using the cloth-dragging method

♂♂, ♀♀, NN ibidem: 8.VI. 2011 collected on vegetation using the cloth-dragging method

♂, ♀, NN ibidem: 9.VI.2011 collected on vegetation using the cloth-dragging method

♂♂, ♀♀, NN ibidem: 15.VI.2011 collected on vegetation using the cloth-dragging method

♂♂, ♀♀, NN ibidem: 3.VII.2011 collected on vegetation using the cloth-dragging method

♂♂, ♀♀, NN ibidem: 4.VII.2011 collected on vegetation using the cloth-dragging method

♂♂, ♀♀, NN, LL ibidem: 2.VIII.2011 collected on vegetation using the cloth-dragging method

♂, ♀♀, NN, LL ibidem: 3.VIII.2011 collected on vegetation using the cloth-dragging method

♀♀, N ibidem: 13.IX.2011 from *Canis lupus familiaris*

**20. *Ixodes trianguliceps* Birula, 1895**

♂♂, ♀♀ Rijeka (Tovornik 1984)

♂♂, ♀♀ Split (Tovornik 1984)

♂♂, ♀♀ Kozjak mountain: Malačka (Tovornik 1984)

NN, LL ibidem: 29.IV. 1974 from *Apodemus mystacinus* (Tovornik 1988b)

♂♂, ♀♀ Velebit mountain (Tovornik 1984)

L ibidem: Veliki Zavižan 28.IX. 1975 from *Sorex araneus* (Tovornik 1988b)

♀ ibidem: Crni padež 1.VIII. 1975 from *Clethrionomys glareolus* (Tovornik 1988b)

L ibidem: Baške Oštarije 11.VIII. 1968 from *Apodemus sylvaticus* (Tovornik 1988b)

L ibidem: Predzid (pod Ćelavcem) 1.VIII.1975 from *Apodemus sylvaticus* (Tovornik 1988b)

♂♂, ♀♀ Istra: Učka mountain (Tovornik 1984)

LL ibidem: 12.VII. 1969 from *Apodemus flavicollis* (Tovornik 1988b)

♂♂, ♀♀ Gorski Kotar: Risnjak mountain (Tovornik 1984)

L ibidem: 9.VIII. 1967 from *Sorex alpinus* (Tovornik 1988b)

♀, L ibidem: 1.IX. 1978 from *Clethrionomys glareolus* (Tovornik 1988b)

♀ Velika Kapela mountain: Razvala 16.VIII. 1968 from *Clethrionomys glareolus* (Tovornik 1988b)

LL Lika: Plitvice, Crna rijeka 12.VIII. 1968 from *Apodemus sylvaticus* (Tovornik 1988b)

LL ibidem: 12.VIII. 1968 from *Apodemus flavicollis* (Tovornik 1988b)

N ibidem: Mukinje 29.V. 1977 from *Clethrionomys glareolus* (Tovornik 1988b)

♀ ibidem: 29.V. 1975 from *Sorex araneus* (Tovornik 1988b)

♀, N, L Medvednica mountain: Sljeme 25.VI. 1975 from *Apodemus flavicollis* (Tovornik 1988b)

N Vukomeričke gorice: Prkovec 19.VII.1974 from *Apodemus flavicollis* (Tovornik 1988b)

N ibidem: 19.VII. 1974 from *Apodemus agrarius* (Tovornik 1988b)

♀♀ Peščenica-Vratovo 5.X. 1977 from *Apodemus flavicollis* (Tovornik 1988b)

N, LL ibidem: 28.X. 1976 from *Sorex araneus* (Tovornik 1988b)

♀ Križevci, Stara Ves 20.IV.1963 from *Apodemus sylvaticus* (Tovornik 1988b)

♀ Peščenica-Vratovo 26.–27.IV. 1984 from *Apodemus flavicollis* (Tovornik and Matjašič 1991)

N ibidem: 2.VII.1984 from *Apodemus flavicollis* (Tovornik and Matjašič 1991)

NN ibidem: 3.VI. 1984 from *Apodemus flavicollis* (Tovornik and Matjašič 1991)

N ibidem: 1.VIII.1984 from *Sorex araneus* (Tovornik and Matjašič 1991)

♀ ibidem: 20.–24.IX. 1983 from *Apodemus agrarius* (Tovornik and Matjašič 1991)

L Peščenica-Vratovo 29.–30.XI.1983 from *Neomys sp*., (Tovornik and Matjašič 1991)

**21. *Ixodes vespertilionis* Koch, 1844**

♀, N Dubrovnik, Močiljska špilja 31.III.1953 from *Rhinolophus ferrumequinum*, *Rhinolophus hipposideros*, *Myotis myotis*, *Miniopterus schreibersii* and from cave habitats (Tovornik 1990b)

♀, L ibidem: 6.V.1961 from *Rhinolophus ferrumequinum*, *Rhinolophus hipposideros*, *Myotis myotis*, *Miniopterus schreibersii* and from cave habitats (Tovornik 1990b)

♂ Krbavsko polje: 17.IX.1963 cave habitats (Tovornik 1990b)

♀ Cres Island: Petrićevi-špilja Čampari 31.III.1975 from *Rhinolophus ferrumequinum*, *Rhinolophus hipposideros*, *Myotis myotis*, *Miniopterus schreibersii* and from cave habitats (Tovornik 1990b)

♂ Buzet, Rabakova peć, Ročko polje, month: VII.1961 from *Rhinolophus ferrumequinum*, *Rhinolophus hipposideros*, *Myotis myotis*, *Miniopterus schreibersii* and from cave habitats (Tovornik 1990b)

♂♂, ♀♀ Brač Island (Hubálek 2010)

## Discussion

Creating a list of ticks present in the Croatian fauna is somewhat complicated because the territory of Croatia was formerly part of the Kingdom of Yugoslavia and later part of the Socialist Federal Republic of Yugoslavia. Before the disintegration of Yugoslavia, nearly all of the records were simply summarized as being from Yugoslavia and the faunas of the new European states were not differentiated. For instance, Oswald (1940, 1941a) mentioned 23 taxa (species, subspecies and variations) of ticks for Yugoslavia without any further data about the localities where the ticks were collected. Only papers published by Mikačić (1949, 1961, 1963, 1965, 1968, 1969) and Tovornik (1976a, 1976b, 1980, 1984, 1987a, 1987b, 1988a, 1988b, 1990a, 1990b, 1991a, 1991b) Tovornik and Šooš (1976), Tovornik and Brelih (1980), Tovornik and Vesenjak-Hirjan (1988) enabled the creation of a list of ticks present in the Croatian fauna. On the basis of current valid species names, 21 species were registered to occur in Croatia. Dobec et al. (2009) recorded *Dermacentor reticulatus* as a new species for Croatian fauna. However, Mikačić (1968) reported that *Dermacentor pictus* (Hermann, 1804) occurred on horses in the greatest number in the territory of northwestern Croatia. He considered that the name *Dermacentor pictus* was a valid name, while the name *Dermacentor reticulatus* was synonymous. Borčić et al. (1978) used the name *Dermacentor reticulatus* correctly, applying the principle of priority which is commonly accepted in zoological nomenclature. Apanaskevich et al. (2010) determined that *Hyalomma (Hyalomma) detritum* Schulze, 1919 is a synonym for the species *Hyalomma (Euhyalomma) scupense* Schulze, 1919. Because of that, *Hyalomma (Hyalomma) detritum* Schulze 1919 is not included in the list of Croatian tick fauna although it was reported in several papers. Furthermore, seven species *Rhipicephalus annulatus*, *Dermacentor reticulatus*, *Haemaphysalis concinna*, *Haemaphysalis inermis*, *Haemaphysalis parva*, *Rhipicephalus sanguineus* and *Ixodes vespertilionis* were not previously included in the previous compilations of ticks for the Croatian fauna on the website of Fauna Europaea. However, some of these species were recorded from several localities in Croatia (Mikačić 1949, 1965, 1968, 1969, Tovornik and Brelih 1980, Tovornik 1976a, 1976b, 1988a, 1988b, 1990a, 1990b, 1991a, 1991b). Most tick species in Croatia were sampled in the Mediterranean part of the country (Mikačić 1949, 1963, 1965, Tovornik and Šooš 1976, Tovornik and Brelih 1980, Tovornik 1976b, 1988b, Tovornik and Vesenjak-Hirjan 1988, Hassl 2003), whereas in the continental part of Croatia only seven species of ticks were sampled (Mikačić 1968, 1969, Tovornik 1976, Borčić et al. 1978), five of which were recorded in a study carried out in 2011 in the territory of Slavonia and Baranja. During our field samplings, *Ixodes ricinus* and *Dermacentor marginatus* were the most common species. According to Hillyard (1996), the frequent occurrence of *Ixodes ricinus* in the field collections throughout Europe seems to be a general trend. The tick fauna of Mikleuš in Slavonia fully corresponds to the tick fauna in Prekmurje and in central Slovenia (Ploj 2007). In most parts of Europe, *Ixodes ricinus* is a common reservoir and the usual vector of *Borelia burgdorferi* *sensu lato* and a variety of other pathogens infecting humans (Jaenson and Jensen 2007). Finally, new data were obtained on the geographical distribution of *Ixodes ricinus*, *Dermacentor reticulatus*, *Haemaphysalis concinna* and *Ixodes hexagonus* because the specimens of these species had not been found previously in eastern Croatia. Only *Dermacentor marginatus* was noted in 1955 in Baranja, in the territory of eastern Croatia (Mikačić 1968).
